# Early biomarkers for predicting sepsis-induced shock: insights from inflammatory pathways and immune response

**DOI:** 10.3389/fphar.2026.1751781

**Published:** 2026-03-05

**Authors:** Jia Li, Qiufang Zhao, Haiyun Gao, Hongjun Wang, Cong Guo, Xiaoling Feng

**Affiliations:** 1 Department of Emergency Medicine, Hebei Provincial Hospital of Traditional Chinese Medicine, Shijiazhuang, China; 2 Department of Emergency Medicine, Hospital of Traditional Chinese Medicine, Shijiazhuang, China; 3 Department of Pharmacy, Hebei Provincial Hospital of Traditional Chinese Medicine, Shijiazhuang, China; 4 Hebei Provincial Hospital of Traditional Chinese Medicine, Shijiazhuang, China

**Keywords:** diagnostic biomarkers, early biomarkers, immune response, inflammatory pathways, sepsis-induced shock

## Abstract

Severe sepsis-induced shock is one of the most challenging problems in critical care despite the progress made in treatment. Recognizing high-risk patients early on is critical for successful results, and the standard diagnostic approaches to such an ailment fail to identify it prior to shock setting in. Biomarkers have become promising diagnostic, prognostic predictors and treatment surveillance platforms in sepsis in the past few years. This review discusses the significance of biomarkers, e.g., cytokines, chemokines, acute-phase proteins and immune dysfunction markers in the pathogenesis of sepsis-induced shock. Additionally, we investigate the potential of new biomarkers, including microRNAs, circular RNAs, endothelial biomarkers, gene signatures, a combination of multimarker panels and machine learning models to improve the diagnostic and prognostic proficiency. As effective as they may seem, they (biomarkers) create challenges in clinical application, including variability, standardization, cost and regulatory approval. This review discusses future approaches to sepsis biomarker research, focusing on personalized medicine, global availability, and clinical validation to address barriers currently experienced in improving sepsis management worldwide.

## 1 Introduction

Sepsis is a critical clinical syndrome precipitated by a deranged host response to infection, characterized by systemic inflammation and acute organ impairment. Severe manifestations of the disease include sepsis-induced shock, which is by definition recognized through persistent hypotension, tissue hypoperfusion and metabolic derangement despite adequate fluid resuscitation and vasopressor support (Chaithanya and Meshram, 2023). This transition of sepsis to shock is a profound point in the pathophysiology of the disease, which raises mortality rates, and many cases of hospital overall mortality rates exceeded 40% in some intensive care unit (ICU) populations. Sepsis management technologies notwithstanding, early identification of patients at risk for shock is still a daunting problem (Beloborodova and Chernevskaya, 2024).

We use Sepsis-3 terminology in this review to reduce conceptual ambiguity. Sepsis is defined as life-threatening organ dysfunction caused by a dysregulated host response to infection and is operationalized clinically as an acute increase in the Sequential Organ Failure Assessment (SOFA) score. Septic shock is a subset of sepsis in which circulatory and cellular/metabolic abnormalities are profound enough to substantially increase mortality risk; operationally, it includes vasopressor requirement to maintain MAP ≥65 mmHg and serum lactate >2 mmol/L despite adequate fluid resuscitation (Singer et al., 2016). Terms such as “sepsis-induced shock” in older literature are treated here as synonyms for Sepsis-3 septic shock unless otherwise specified.

The target of this review is early prediction of Sepsis-3 septic shock, operationally defined as the need for vasopressors to maintain a mean arterial pressure (MAP) of at least 65 mmHg and a serum lactate level >2 mmol/L despite adequate fluid resuscitation (Singer et al., 2016). The primary intended-use setting is early care, focusing on emergency department triage and initial resuscitation (0–6 h), with a secondary discussion on ICU monitoring (6–24 h and beyond). Table 1 summarizes the key biomarkers in sepsis-induced shock and their clinical significance. A key distinction is between biomarkers interpreted for short-horizon shock escalation (e.g., vasopressor initiation within a defined window) and biomarkers that are primarily prognostic (e.g., late organ failure or long-horizon mortality) and therefore should not be presented as ED-timepoint shock prediction evidence.

**TABLE 1:** Key biomarkers in sepsis-induced shock.

Biomarker	Role	Associated pathways	Clinical significance in sepsis/septic shock	Representative quantitative performance in sepsis (where available)
IL-6 (Interleukin-6)	Pro-inflammatory cytokine	Innate immune activation, acute-phase response, JAK/STAT and NF-κB signalling	Rapidly rises in early sepsis; higher levels correlate with organ dysfunction, septic shock and mortality. Useful for early diagnosis and risk stratification, especially when combined with clinical scores	In an ED cohort of 142 adults (51 sepsis, 46 septic shock, 45 controls), serum IL-6 at admission showed AUROC ≈0.89 for sepsis vs. non-septic controls at a cut-off around 50 pg/mL (sensitivity ≈80%, specificity ≈89), and AUROC ≈0.80 for progression to septic shock; high IL-6 strata had markedly higher 28-day mortality
TNF-α (Tumor necrosis factor-α)	Pro-inflammatory cytokine	NF-κB activation, apoptosis, endothelial activation	Central mediator of early systemic inflammation and microvascular injury. Elevated levels are associated with shock and multi-organ failure but show substantial overlap between survivors and non-survivors	Prospective sepsis and bloodstream-infection cohorts consistently show higher TNF-α in severe sepsis and non-survivors, but single-marker AUROC and cut-off values are rarely reported and generally inferior to IL-6 or PCT; TNF-α is mainly used within multi-marker panels rather than as a standalone test
PCT (Procalcitonin)	Acute-phase reactant	Systemic inflammatory and neuroendocrine response to bacterial toxins	Widely used for bacterial infection and sepsis; supports diagnosis, antibiotic stewardship, and prognosis. Levels correlate with infection severity and treatment response	In mixed ED infection/sepsis cohorts, PCT shows modest prognostic value for 28-day mortality (e.g., AUROC ≈0.59) but stronger performance for bacteremia among ICU septic patients (AUROC ≈0.85), with cut-offs typically in the 0.5–2 ng/mL range depending on setting
CRP (C-reactive protein)	Acute-phase reactant	Hepatic response to IL-6 and other cytokines	Non-specific marker of inflammation; useful to detect infection and follow trends but limited for sepsis risk stratification when used alone	In ED cohorts with sepsis or severe infection, CRP shows weak discrimination for 28-day mortality (AUROC ≈0.55), though ICU studies report better performance for bacteremia prediction (AUROC ≈0.76), still lower than PCT.
IL-10 (Interleukin-10)	Anti-inflammatory/immunoregulatory cytokine	Downregulates pro-inflammatory cytokine production, promotes immunosuppressive phenotypes	Reflects compensatory anti-inflammatory response and sepsis-induced immunosuppression. High or persistently elevated IL-10 is associated with secondary infections and worse outcomes	In bloodstream infection cohorts, IL-10 has shown better diagnostic performance than CRP and IL-6 for sepsis/bacteremia discrimination (e.g., AUROC around 0.86, sensitivity and specificity both >0.75), particularly when combined with PCT.
sTREM-1 (soluble triggering receptor expressed on myeloid cells-1)	Amplifier of innate immune activation	Myeloid cell activation, amplification of pro-inflammatory cytokine release	Elevated in bacterial sepsis and septic shock; considered a promising early marker for differentiating infectious from non-infectious SIRS and for predicting organ failure	Meta-analyses of sTREM-1 in sepsis report pooled AUROC values around 0.80–0.85, with sensitivity and specificity generally in the 0.75–0.85 range for sepsis vs. non-septic SIRS, suggesting good but not definitive diagnostic accuracy

In order to decrease conflation between studies and endpoints, biomarkers are addressed according to clinical intent and timing of decisions: (i) diagnostic biomarkers, which help characterize the sepsis/infection phenotype at presentation; (ii) early risk-prediction biomarkers, which aim to determine patients who will require vasopressors and develop shock within a set time-period; (iii) prognostic biomarkers, which estimate mortality or organ-failure risk after sepsis or shock has already been established; and (iv) monitoring biomarkers, which track treatment response and trajectory over time and may support escalation or de-escalation decisions.

The pathogenesis of shock from sepsis is multi-factorial and involves crosstalk of pro-inflammatory cytokines, immune cell dysfunction, endothelial injury, coagulation abnormalities and mitochondrial dysfunction. In the initial stages of the disease, an exaggerated hyperinflammatory state resulting from high levels of cytokines (IL-6 and TNF-α) induces endothelial damage, increased vascular permeability, and severe tissue injury. That is followed by an immunosuppressive phase, commonly known as “immunoparalysis,” whereby the host cannot fight secondary infections, increasing the morbidity and mortality of the patients even further (Chaithanya and Meshram, 2023).

Traditionally, clinical indicators such as heart rate, blood pressure, and lactate levels have been used to evaluate the severity. These, however, are usually only valuable for the presentation of late disease and have little predictive effect. Molecular biomarkers have become important in nucleating early disruptions of inflammatory and immunological pathways, generating predictive foresight well before overt clinical manifestations appear (Beloborodova and Chernevskaya, 2024). These biomarkers, such as the cytokines, acute-phase proteins, immunocell markers, and genomic signatures, are of interest for the increasing study on early diagnosis, risk stratification and monitoring of treatment responses in a sepsis-induced shock.

This review aims to provide a detailed overview of early biomarkers of sepsis-induced shock and their role in inflammatory pathways and immune response. We will investigate how the biomarkers reflect the pathobiology of sepsis, the accuracy of diagnosis through their use, and their possibility for use in guiding clinical decision-making. In addition, we discuss the emerging biomarkers discovered through omics, including microRNAs, gene signatures and proteomic signatures, and how these markers have been incorporated into multi-marker panels of higher clinical use (Yuan et al., 2019). Finally, we shall consider the difficulties in incorporating these biomarkers in routine clinical practice concerning standardization, cost, and turnaround time.

### 1.1 A time- and decision-oriented framework for biomarker use in septic shock risk

Translation of sepsis biomarkers into clinical practice often fails because many candidates are reported as generic severity correlates rather than as predictors of specific actions within defined time windows. Based on this review, we organize evidence around three functional decision nodes: (1) ED/early ward triage (0–3 h) to identify sepsis and imminent risk of escalation; (2) early resuscitation (3–6 h) when decisions about vasopressor initiation and ICU admission are made; and (3) ICU monitoring (6–24 h and beyond) for prognostication and therapeutic-response tracking. Each section therefore interprets biomarkers in relation to their kinetics, endpoints, and the concrete management choices they can reasonably inform (e.g., intensified monitoring, earlier ICU transfer, hemodynamic assessment, antibiotic stewardship, or enrollment in immunomodulatory trials).

Because clinical applicability depends strongly on timing, Table 2 provides a decision-level synthesis of candidate markers across three care phases: ED triage (0–3 h), early resuscitation (3–6 h), and ICU monitoring (6–24 h and beyond). This schema maps biomarkers to plausible bedside actions (e.g., escalation/ICU referral, intensity of hemodynamic surveillance, preparedness for vasopressor support, antibiotic stewardship, and prognostic enrichment) while acknowledging that biomarker-driven clinical pathways remain incompletely validated.

**TABLE 2:** Biomarkers mapped to time-critical decision points in sepsis and septic shock risk.

Decision window	Bedside decision to support	Biomarkers discussed in this review (examples)	Intended role (be explicit)	Practical note (timing/limits)
0–3 h (ED/early ward triage)	Confirm infection/sepsis; decide early escalation/monitoring intensity	PCT, CRP, IL-6, IL-8 (with clinical scores/lactate)	Diagnosis support + early risk stratification	Single markers are imperfect; interpret alongside clinical scores; prioritize tests with rapid turnaround
3–6 h (early resuscitation)	Anticipate vasopressor/ICU need; trigger early hemodynamic assessment	IL-6 (where validated), endothelial/glycocalyx markers (e.g., syndecan-1), cardiac strain markers (NT-proBNP)	Prediction of deterioration/escalation risk (where validated)	Requires studies anchored to a defined endpoint (e.g., vasopressor requirement within a window); thresholds vary by cohort
6–24 h+ (ICU monitoring/prognosis)	Prognostication; monitor trajectory; guide stewardship/adjunctive trials	Serial PCT/CRP trends, NT-proBNP, cytokine/chemokine panels, endothelial injury markers	Monitoring + prognosis; immune-domain endotyping	Trajectory/serial change often more informative than a single value; interpret with renal function, comorbidities

### 1.2 Biomarker kinetics and optimal sampling strategy

The clinical usefulness of biomarkers in sepsis is time dependent. Early inflammatory mediators (e.g., interleukin-6 and tumor necrosis factor-alpha) peak sooner, whereas evidence of endothelial injury, coagulation activation, and immune suppression often evolves more gradually over hours to days. Consequently, single time-point assessment may be insufficient; serial sampling (baseline and later at 6–24 h) can improve risk stratification. In addition to static values, dynamic measures (e.g., lactate clearance, procalcitonin kinetics, trajectory-based scoring systems) provide a more realistic picture of the physiologic response to resuscitation and the risk of imminent deterioration. Based on this, biomarker classes are interpreted by (i) the latest time when actionable interpretation is plausible, (ii) whether serial monitoring is recommended, and (iii) the incremental value of trajectories relative to baseline values.

## 2 Literature search and article selection

### 2.1 Search strategy and sources

PubMed/MEDLINE, Embase, and Web of Science (January 2000–September 2025) were searched, limiting to the records in English language, and with particular attention to the translational and clinical validation studies published after 2020. Search terms were designed to include the concepts of sepsis/shock with biomarker categories and endpoints with clinical implications [e.g., vs. initiation of vasopressors, episode of hypotension, lactate concentration, changes in Sequential Organ Failure Assessment (SOFA) score, and intensive care unit admission] (Tricco et al., 2018).

### 2.2 Screening and eligibility

Relevance screening was conducted based on (i) inclusion of (a) populations by Sepsis-3 criteria of sepsis or septic shock (population) and (b) studies that undertake early prediction or risk stratification and/or include time-sampling diagnostic or prognostic performance metrics (area under the receiver operating characteristic curve, sensitivity, specificity, hazard ratio) when applicable. Full-text articles were reviewed on the basis of specific endpoint definitions and sampling time points. The studies with no clear endpoint definitions or clear sampling times were excluded.

### 2.3 Data extraction and synthesis

Within each biomarker category, we extracted the clinical setting (emergency department, general ward, intensive care unit), timing of sampling relative to the endpoint, assay type, and external validation status. Evidence was synthesized into a structured narrative consistent with the Scale for the Assessment of Narrative Review Articles (SANRA) principles (Baethge et al., 2019) and mapped to actionable decision nodes (0–3 h, 3–6 h, 6–24 h).

Potentially eligible records included clinical observational cohort studies, diagnostic or prognostic accuracy studies, randomized trials assessing biomarker-based decision-making, and well-conducted translational studies with a specified endpoint. Studies, which reported (i) timing of sampling, (ii) one of the explicit clinical endpoints (i.e., vasopressor requirement, shock onset within defined window, changes in SOFA score, or mortality), and (iii) quantitative measures of performance (when they were available) (i.e., AUROC, sensitivity, specificity, hazard ratios) were of higher priority. Unclear endpoints, lack of reporting of the timing of sampling or an assertion that was not consistent with the design of the study were considered exclusion criteria.

### 2.4 Review type statement

This article is a narrative review with a structured literature search and a uniform evidence-extraction rubric (structured narrative review). It is not intended as a full scoping review with complete PRISMA flow accounting; therefore, we focus on transparent reporting of search sources, eligibility logic, and endpoint/time-window alignment rather than exhaustive study enumeration.

Regardless of identification of hundreds of candidate biomarkers, translation into better patient outcome has never yet been achieved. The most frequent barriers include small, single-centre cohorts; non-homogenous or not well defined endpoints; inadequate consideration of sampling time; and absence of interventional studies that assess biomarker directed mechanisms as opposed to straightforward associative studies. Therefore, the current review particularly highlights research studies that offer definite timeframes and actionable conclusions like vasopressor demand, ICU hospitalization, or death. It also points out the gaps where the evidence is inadequate to be put into clinical practice. By directly relating biomarkers to tangible points of decision-making we hope to outline those signals that can be cautiously translated into practice and those that can largely be considered exploratory.

### 2.5 Uniform evidence-extraction framework across biomarker classes

We applied a uniform evidence-extraction framework across all biomarker classes to reduce interpretive heterogeneity and to distinguish association from clinically actionable prediction within defined decision windows. For each study we captured: design/setting, population, sampling time (relative to presentation), comparator/reference standard, endpoint and prediction horizon, and reported performance metrics (e.g., AUROC, sensitivity, specificity). Whenever a metric reflects diagnostic discrimination (e.g., sepsis vs. non-septic controls) rather than short-horizon shock escalation, it is explicitly labeled as diagnostic/biological plausibility evidence rather than early shock prediction at presentation.

## 3 Inflammatory pathways in sepsis

The sepsis-induced shock pathogenesis is dependent on the inflammatory response. On detection of pathogen-associated molecular patterns (PAMPs) or damage-associated molecular patterns (DAMPs), the immune cell pattern recognition receptors (PRRs) are switched on, which initiate a sequence of signalling events resulting in the release of pro-inflammatory cytokines. This early hyperinflammatory reaction is indeed a protective measure set against infection. However, in sepsis, this response gets dysregulated, and there is diffuse tissue injury, vascular instability, and organ dysfunction, all leading to shock (Barichello et al., 2022).

### 3.1 Cytokines in sepsis-induced shock

Cytokines play a cardinal role in the inflammatory response in sepsis (Wong, 2019). They are small proteins that the immune cells produce that govern immune cell trafficking, activation and survival. At the beginning of sepsis, a cytokine storm is observed where massive release of pro-inflammatory cytokines, including interleukin-6 (IL-6), tumor necrosis factor-α (TNF-α) and interleukin-1 beta (IL-1β). These cytokines commence the recruitment of immune cells to the infected wound and activation of endothelial functions and vasodilation, hence increased vascular permeability and hypotension–markers of septic shock (Faix, 2011).

### 3.2 Study design and setting (ED/ward/ICU; prospective vs. retrospective)

The available evidence mostly relates to observational cohorts in emergency departments (EDs) and intensive care units (ICUs) that involve early cytokine tracking. A single-centre, prospective ED study using a Sepsis-3 workflow measured interleukin-6 (IL-6) at ED presentation and evaluated its diagnostic and prognostic value (Song et al., 2019). Serial sampling within the first 24 h (at arrival and prespecified early time intervals) captured IL-6 kinetics in a separate prospective ED cohort (Macdonald et al., 2014). Interleukin-8 (IL-8) has been evaluated as a risk-stratification biomarker in critically ill patients with sepsis, with derivation and external validation across two centres (Anderson et al., 2019).

### 3.3 Population characteristics (adult vs. pediatric; severity spectrum; inclusion criteria)

The majority of the cohorts involve skewed adult sepsis populations towards more extreme states. The Sepsis-3 ED study was a randomized controlled trial involving adults aged 18 years or older that met sepsis criteria with a qSOFA/infection/SOFA score of two or more and compared patients with sepsis and septic shock (Song et al., 2019). The serial-sampling ED cohort differentiated between uncomplicated sepsis and severe sepsis or septic shock; in the latter, the majority of severe cases occurred as a result of severe sepsis or septic shock (Macdonald et al., 2014). In the multicentre IL-8 threshold study, the analyses were further stratified into immunocompromised versus immunocompetent (Anderson et al., 2019).

### 3.4 Sampling window and kinetics-relevant timing (recognition; 0–3 h, 3–6 h, 6–24 h; single vs. serial)

Timing is a major source of heterogeneity in cytokine measurement. In a Sepsis-3 ED cohort, the initial IL-6 sample was obtained within ∼6 h of clinical recognition of septic shock, with follow-up IL-6 measurements later in the clinical course (e.g., near discharge or outcome ascertainment) (Song et al., 2019). In a separate ED serial-sampling cohort, IL-6 was measured at ED arrival (baseline), 1–2 h, 3–6 h, and 12–30 h, illustrating how sampling windows can materially change observed discrimination and associations with outcomes (Macdonald et al., 2014). In a multicentre ICU-adjacent cohort, cytokine sampling was timed as close as possible to ICU bed request and within 24 h of ICU admission, capturing early critical care physiology rather than ED-arrival kinetics (Anderson et al., 2019).

### 3.5 Comparator/reference standard (Sepsis-3 shock definition; lactate; SOFA/qSOFA; other tools)

Across the field, septic shock definitions rely on hemodynamics and lactate, but operational details vary by protocol and time window. In a Sepsis-3 ED cohort, IL-6 at presentation was compared with established clinical predictors such as lactate, SOFA, and APACHE II in relation to severity and 28-day mortality (Song et al., 2019). In a multicentre enrichment study, septic shock was operationalized using vasopressor requirement and serum lactate >2 mmol/L at ICU admission, consistent with Sepsis-3 criteria (Anderson et al., 2019). Broader definitions and consensus details are provided in the Sepsis-3 and septic shock definition literature (Singer et al., 2016; Shankar-Hari et al., 2016).

### 3.6 Endpoints and prediction horizon (transition to vasopressor-dependent shock; time-to-shock; mortality)

Many cytokine studies conflate severity discrimination (sepsis vs. septic shock) with long-horizon mortality prediction. In a Sepsis-3 ED cohort, IL-6 discriminated septic shock (AUC ≈0.80) and an IL-6 cut-off (≈349 pg/mL) aligned with both shock discrimination and separation of 28-day survival curves (Song et al., 2019). Mechanistic and clinical data have linked elevated IL-8 to shock biology and lactate elevations in sepsis (Hack et al., 1992). However, in a multicentre cohort IL-8 was primarily evaluated as a 30-day mortality risk marker in immunocompetent patients, rather than as a time-to-shock predictor (Anderson et al., 2019).

### 3.7 Validation approach (internal vs. external; calibration/transportability)

External validation remains limited for many cytokine biomarkers in real-world bedside workflows. For example, the Sepsis-3 ED IL-6 cohort was single-centre and explicitly called for multi-centre verification (Song et al., 2019). In contrast, the multicentre IL-8 enrichment study included derivation in one cohort and validation in a second, supporting better transportability than single-centre analyses; however, validation still occurred in similar high-resource ED-to-ICU settings (Anderson et al., 2019).

**FIGURE 1:**
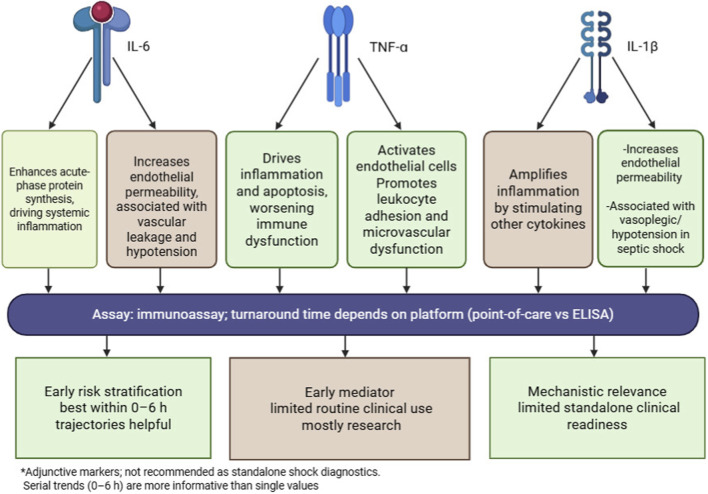
Roles and clinical readiness of key pro-inflammatory cytokines (IL-6, TNF-α, IL-1β) in early sepsis. The figure summarizes major pathophysiologic effects, typical assay modality (immunoassay; turnaround varies by point-of-care vs. ELISA platforms), and practical clinical interpretability. Cytokines are best interpreted as adjunct markers; early trajectories within 0–6 h may add value over single measurements.

Figure 1 shows the cytokine storm, highlighting key cytokines like IL-6, TNF-α, and IL-1β, and their effects on immune response and endothelial function.

#### 3.7.1 Interleukin-6 (IL-6)

One of the most widely investigated cytokines in sepsis is IL-6, which has a pro- and anti-inflammatory function. It is quickly produced upon PRR activation and works via the Janus kinase/signal transducer and activator of transcription (JAK/STAT) pathway (Fraunberger and Walli, 2007). High levels of IL-6 are associated with the severity of disease, organ dysfunction and mortality in septic patients. Its early increase suggests hemodynamic instability and septic shock development, hence a proper prognostic indication in critical care (Faix, 2011). In a prospective study using Sepsis-3 definitions (n = 142; 51 sepsis, 46 septic shock, 45 controls), Song et al. reported that IL-6 measured at emergency department presentation discriminated sepsis from controls and also identified patients with septic shock. Using an IL-6 cut-off of 348.9 pg/mL, IL-6 showed utility for predicting septic shock, and higher IL-6 levels were associated with increased 28-day mortality among septic shock patients (Song et al., 2019). These findings support IL-6 in being used as an early sign of severity and escalation in comparison with a predetermined endpoint over a given period.

Although often discussed alongside inflammatory mediators in sepsis workups, procalcitonin (PCT) is an acute-phase host-response biomarker and is therefore discussed under acute-phase proteins.

#### 3.7.2 Tumor necrosis factor-alpha (TNF-α)

TNF-α is an early released cytokine in infection. It increases endothelial adhesion molecule expression, increases vascular permeability, and causes apoptosis of immune and parenchymal cells. High levels of TNF-α correlate with poor prognosis, quick advancement to a state of shock, and high death rates, despite complications in measurement because of the short half-life of TNF-α as a critical cytokine in sepsis pathophysiology (Jekarl et al., 2019). Tumor necrosis factor alpha (TNF -a) levels are typically higher in cases of severe sepsis and non-survivors of prospective cohorts of sepsis and bloodstream infection. However, because of a very early peak of TNF -alfa and a lot of overlap between levels in outcome groups it is not common practice to establish independent threshold values or area under the receiver operation curve (AUROC); compared to interleukin-6 (IL-6) or procalcitonin (PCT), TNF -alfa usually performs poorly. TNF -a, therefore, can best be viewed as a risk stratification and endotyping adjunct within the inflammatory spectrum best used as a multiple parameter system, as opposed to use as a single diagnostic or shock-forecasting measure.

**FIGURE 2:**
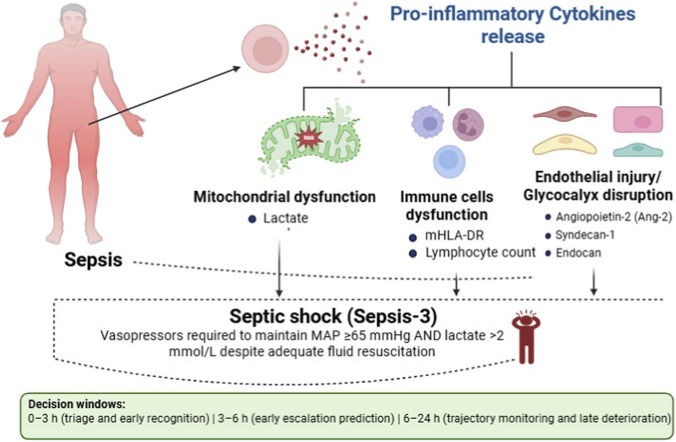
Conceptual pathway linking sepsis pathobiology to septic shock (Sepsis-3) and representative biomarker classes across early decision windows. Pro-inflammatory cytokine release contributes to mitochondrial dysfunction (e.g., lactate), immune dysregulation (e.g., mHLA-DR, lymphocyte count), and endothelial/glycocalyx injury (e.g., Ang-2, syndecan-1, endocan), which can converge on septic shock. Decision windows shown: 0–3 h (triage/recognition), 3–6 h (early escalation prediction), and 6–24 h (trajectory monitoring/late deterioration). Abbreviations: Ang-2, angiopoietin-2; mHLA-DR, monocyte HLA-DR.

Figure 2 illustrates the progression from sepsis to shock, showing the interactions between pro-inflammatory cytokines, immune cell dysfunction, endothelial injury, coagulation abnormalities, and mitochondrial dysfunction.

Table 2 summarizes the biomarkers (e.g., IL-6, TNF-α, PCT, CRP) with columns for their roles, associated pathways, and clinical significance.

### 3.8 Chemokines and their role in sepsis

Chemokines refer to a subgroup of cytokines that tend to cue to migration of immune cells to the site of infection. They have a vital role in modulating the immune response, they are crucial in early sepsis days. Chemokines, including IL-8, CCL5 and CXCL10, are involved in attraction to the infection site of neutrophils, monocytes, and T cells (Sasi et al., 2023).

**FIGURE 3:**
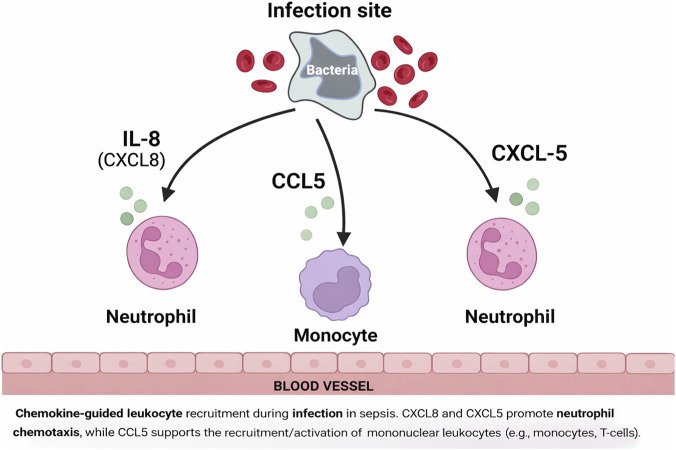
Chemokine-guided leukocyte recruitment during infection in sepsis.

A visual representation of chemokine action, illustrating how IL-8, CCL5, and CXCL10 recruit immune cells to infection sites (Figure 3).

#### 3.8.1 IL-8 (CXCL8)

IL-8 is a strong chemokine that attracts neutrophils to a site of infection. High levels of IL-8 are known to correlate with a worsening of disease and organ failure in sepsis. It is an early severity and prognostic marker in sepsis (organ dysfunction and short-term mortality), but there is less consistency in the evidence supporting IL-8 as an independent predictor of shock onset or vasopressor requirement, and this depends on the time of measurement and endpoint (Cioara et al., 2016). Zhang et al. (2024) indicated that the serum IL-8 at 1 h of emergency department admission could predict 28-day mortality in elderly sepsis patients with an outcome of an AUROC of 0.701 and sensitivity of 74.5% and specificity of 63.4% at an outcome cut-off of 14.497 pg/mL.

#### 3.8.2 C-C motif ligand 5 (CCL5)

CCL5 is implicated in T-cells and monocytes recruitment during inflammation. It has demonstrated a good diagnostic performance for sepsis, with early distinction of sepsis from other inflammatory disorders. Its high levels are associated with a bad prognosis, suggesting its potential biomarker ability in the diagnosis of sepsis. Jekarl et al. (2019) reported an AUROC of 0.887 for C-C motif ligand 5 (CCL5/RANTES) measured on the day of sepsis diagnosis in ICU patients to discriminate sepsis from healthy controls, with sensitivity 90.1% and specificity 76.3% at a cut-off of 179.6 pg/mL.

#### 3.8.3 C-X-C motif chemokine 10 (CXCL10)

CXCL10 has a central role in attracting activated T cells and monocytes to the focus of infection. It has been characterized as a promising candidate biomarker for sepsis diagnosis, for which it has been associated with mortality in several cohorts, but performance varies by timing and case-mix. The CXCR3/CCR5 axis, by way of dysregulation of ligands such as CXCL9 and CXCL10, is an important aspect during early sepsis and a potential target for an early diagnosis (Sardesai et al., 2021).

In sepsis, CXCL10 has been linked to severity and mortality in several cohorts, but its apparent performance varies substantially by sampling time, case-mix, and endpoint. definition. A key limitation is that CXCL10 is not specific to sepsis and may be elevated across multiple inflammatory and infectious conditions; therefore, its most plausible near-term role is within multi-marker immune-response panels (or endotype classification) rather than as a standalone diagnostic test. Clinically, CXCL10 is best framed as a marker that may support immune-domain risk stratification (prognosis/endotyping) rather than direct early “shock prediction” unless validated against a vasopressor/time-window endpoint.

### 3.9 Acute-phase proteins and their diagnostic value

Acute-phase proteins (APPs) are biomarkers that increase following inflammation. They are routinely used in sepsis diagnosis and treatment because they react quickly to infection and can indicate systemic inflammation. Important acute-phase proteins in the case of sepsis include C-reactive protein (CRP), procalcitonin (PCT) and fibrinogen (Chowdhury, 2024).

#### 3.9.1 C-reactive protein (CRP)

CRP is an acute-phase protein that increases during systemic inflammation. Although not specific to PCT, CRP is often part of other biomarkers in detecting and managing sepsis. High CRP levels correlate with the severity of an infection and are frequently employed to evaluate the results of antimicrobial therapy (Kim and Choi, 2020). Mohanty and Bhuyan (2023) reported an AUROC of 0.949 for serum C-reactive protein (CRP) measured on day 0 at admission in predicting sepsis (Sepsis-3) versus non-septic controls, with 89.1% sensitivity and 91.3% specificity at a cut-off ≥12 mg/L.

#### 3.9.2 Procalcitonin (PCT)

Procalcitonin (PCT) is a host-response acute-phase biomarker that rises in systemic bacterial infection and has been widely evaluated for sepsis diagnosis, severity stratification, and treatment monitoring; its kinetics and interpretability depend on the clinical context and serial trends. PCT is a 116-amino-acid precursor of calcitonin which is typically produced in thyroid C-cells at extremely low circulating levels. However, in systemic bacterial infection, PCT rises rapidly in several extra-thyroid tissues (in response to bacterial endotoxins and inflammatory mediators (e.g., IL-6, TNF-a, IL-1b)) and is usually found to begin within several hours and reach its peak within the first 24–48 h depending on the severity of the disease. Clinically, PCT should be clinically supported as a diagnostic supplement to bacterial infection and a marker of monitoring/antibiotic stewardship; evidence that PCT is a good predictor of future septic shock is mixed and heavily relies on what is defined as such (ex. whether it is required to use vasopressors within a given time frame), what the baseline is, and at what time it was tested. Representative studies include (Assicot et al., 1993; D’Onofrio et al., 2022; Goyal et al., 2024; Liu et al., 2020; Varga et al., 2025; Zhang et al., 2024; Zhou et al., 2023; Zhou et al., 2024).

On a quantitative basis, on mixed emergency department infection/sepsis cohorts, admission PCT tends to only modestly differentiate short-term mortality (AUROC 0.59), but can higher in the case of bacteremia in ICU septic patients (AUROC 0.85–0.5), with typical cut-offs of 0.5–2 ng/mL, depending on the clinical environment and time of sampling, (Park et al., 2013).

Notably, PCT gives the most informative results when interpreted in a serial (clearance or trajectory) fashion to aid in monitoring and antibiotic stewardship and not at all as a predictor of future septic shock. Such review concludes that in the context discussed here, PCT is mostly viewed as an early infection/sepsis support marker, and a trajectory marker, and not as a shock-prediction device on its own.

#### 3.9.3 N-terminal prohormone of brain natriuretic peptide (NT-proBNP)

NT-proBNP is a marker of cardiac dysfunction and has become a predictor of mortality in septic shock. High levels of NT pro-BNP are linked to a weakened cardiac function and are prognostic for sepsis, especially in subjects with underlying cardiovascular comorbidities (Rogić et al., 2017). NT-proBNP is a marker of myocardial stress and cardiomyopathy related to sepsis. High concentrations are linked to a higher level of shock severity, need more vasopressor, and unfavorable clinical outcomes, but its levels are heavily influenced by renal functionality, age, and pre-exist cardiac pathology.

In a meta-analysis of 3,508 patients with sepsis, it was identified that a threshold of about 4,000 pg/mL is the most effective in predicting short-term mortality (AUROC = 0.79, Sensitivity = 0.73, Specificity = 0.79). Conversely, individual studies provide an approximate 0.63 to 0.87 range of the AUROC at a 400 to 13,600 pg/mL range. Therefore, NT-proBNP is to be viewed as mostly a prognostic and monitoring biomarker of cardiac dysfunction, and not as an early shock-predictive test by itself or diagnostic test of sepsis. Table 3 enlists acute-phase proteins such as CRP, PCT, and NT-proBNP, with details on their diagnostic uses and limitations.

**TABLE 3:** Acute-phase proteins and diagnostic value.

Acute-phase protein	Diagnostic use	Clinical significance in sepsis/septic shock	Limitations	Representative quantitative performance in sepsis (where available)
CRP (C-reactive protein)	Detecting systemic inflammation, monitoring suspected sepsis, and tracking response to therapy	Rises in bacterial infection, tissue injury and inflammatory states. Serial declines can support treatment response and infection control	Low specificity: elevated in many non-infectious inflammatory conditions (trauma, autoimmune disease, surgery), and single values perform poorly for risk stratification	ED sepsis cohort: AUROC ≈0.55 for 28-day mortality prediction (weak prognostic value). ICU sepsis cohort: AUROC ≈0.76 for bacteremia among septic patients (still inferior to PCT)
PCT (Procalcitonin)	Differentiating bacterial from viral or non-infectious causes and supporting sepsis diagnosis	More specific than CRP for bacterial infection; higher levels and slower clearance are associated with severity, shock and mortality. Used to guide initiation and de-escalation of antibiotics	Can be elevated in major surgery, trauma or cardiogenic shock; optimal cut-offs vary by population and timing, and cost can limit routine use	For 28-day mortality in ED infection/sepsis, AUROC ≈0.59; for bacteremia prediction in ICU septic patients, AUROC ≈0.85 with cut-offs around 0.5–2 ng/mL depending on cohort
NT-proBNP (N-terminal pro-B-type natriuretic peptide)	Assessing cardiac dysfunction and short-term mortality risk in sepsis	Reflects myocardial strain and sepsis-associated cardiomyopathy; higher levels correlate with shock severity, vasopressor requirements and adverse outcomes	Strongly influenced by renal function, age and pre-existing heart disease; not specific to sepsis and should be interpreted with clinical context	Meta-analysis of 3,508 sepsis patients identified ∼4,000 pg/mL as an optimal NT-proBNP cut-off for short-term mortality (AUROC ≈0.79; sensitivity ≈0.73, specificity ≈0.79), with individual studies reporting AUROC values between ∼0.63 and 0.87 at cut-offs ranging 400–13,600 pg/mL
Albumin	Assessing nutritional status, chronic inflammation and liver synthetic function	Hypoalbuminemia is frequent in sepsis and correlates with capillary leak, severity of illness and mortality; often incorporated into prognostic scores	Very non-specific; strongly affected by chronic liver disease, nephrotic syndrome, malnutrition and fluid shifts, and changes slowly with acute interventions	Observational sepsis cohorts show that lower admission albumin independently predicts mortality, but albumin alone rarely achieves AUROC >0.7 and is not routinely reported as a stand-alone ROC metric; it is usually embedded within composite scores
Fibrinogen	Assessing coagulation status and risk of DIC in sepsis	Acute-phase reactant that increases in early inflammatory and pro-thrombotic states; low levels later may indicate consumption in overt DIC.	Levels are influenced by pregnancy, liver disease, anticoagulant therapy and chronic inflammatory states; dynamic changes may be more informative than single values	In sepsis, fibrinogen is primarily evaluated as part of DIC scores or broader coagulation panels; few studies report stand-alone AUROC for sepsis diagnosis or mortality, and available data suggest only moderate discrimination when used alone

### 3.10 Endothelial dysfunction in sepsis-induced shock

Endothelial dysfunction is a signature feature of sepsis-induced shock and implicates the appearance of vasodilation and capillary leak, and subsequent tissue hypo-perfusion. Markers of endothelial activation and injury are of growing interest in studying their diagnostic and prognostic functions (Trzeciak et al., 2020).

### 3.11 Structured synthesis (uniform evidence-extraction rubric)

The existing evidence base in favor of utilizing endothelial dysfunction biomarkers within the framework of sepsis-related shock is mostly a reflection of observational cohorts that came into being within the emergency department (ED) and intensive care unit (ICU) setting. Much of the heterogeneity that is evident in studies is explained by differences in sampling windows and in the definitions of clinical outcomes used.

Regarding study design and the setting, researchers have compared the endothelial biomarkers during the cases of early presentation in the ED or those pre-existing populations of patients with septic shock in the ICU. As an example, baseline levels and 24-h levels of soluble vascular endothelial growth factor-A (VEGF-A) and soluble fms-like tyrosine kinase-1 (sFLT-1) were measured in a prospective ED cohort of patients with suspected infection (Shapiro et al., 2008). On the contrary, the serial measurement of angiopoietin-2 was carried out during the ICU course of the mechanically ventilated patients with septic shock (van der Heijden et al., 2009). The studied groups are mainly adult cohorts and show a preference towards extreme morbidity, such as sepsis-induced hypoperfusion and septic shock. In addition, analyzed samples across studies differ very widely in the temporal distribution (single draws in early, serial sampling across multiple hours to days) and this may have confounded observed studies.

In regard to comparators and reference values, investigators tend to scale endothelial biomarkers to both conventional definitions of shock with clinical tools as well as different forms of severity scales, including lactate concentration, vasopressor exposure, the Sequential Organ Failure Assessment (SOFA) score, Acute Physiology and Chronic Health Evaluation II (APACHE) and longitudinal organ dysfunction patterns (Shapiro et al., 2008; Mihajlovic et al., 2014; van der Heijden et al., 2009). Most papers confound discrimination of degree (between sepsis and septic shock), progression of organ failure, such as multiple organ dysfunction syndrome (MODS), and all-cause mortality, as opposed to trying to predict the actual time to vasopressor-dependent shock. The measurement of endocan levels within 24 h of the onset of sepsis or ICU admission has been shown to be linked to organ failure, development of MODS and 28-day mortality (Mihajlovic et al., 2014). On the same note, the diagnosis of septic shock, the burden of vasopressor use, and later clinical outcomes of ED cohorts have also been associated with the level of syndecan-1, particularly with baseline measurements (T0) and 6 h (T6) measurements (Puskarich et al., 2016; Saoraya et al., 2021).

Validation is one of the most critical constraints of the existing literature. Despite a meta-analytic synthesis showing angiopoietin-2 to be a predictor of mortality in adult patients with sepsis (Zhuo et al., 2024), most of the studies on the individual biomarkers use single-center or internally-validated cohorts. The issue of multicenter external validation across different clinical locations and patient care trajectories is therefore still of utmost importance before these biomarkers can be safely taken to bedside practice.

#### 3.11.1 Endocan (ESM-1)

Endocan is a dermatan sulfate proteoglycan released by endothelial cells in response to pro-inflammatory cytokines. High endocan has been linked to high vascular permeability, septic shock and poor prognosis. Its capacity to reflect endothelial dysfunction is an important trait, as a marker of sepsis management (Sandquist and Wong, 2014). In the sepsis setting, endocan has been mainly studied as an endothelial activation and injury biomarker, and it has been found that high endocan concentrations are associated with higher capillary leakage, more severe dysfunction of organs, and unfavorable clinical outcome; nevertheless, the strength of these correlations and the best chosen cutoff remain inter-study diverse, presumably due to variations of the cohort and time sampling. Endocan then must be considered as a risk stratifying and longitudinal monitoring auxiliary tool (especially in combination with other endothelial and inflammatory biomarkers) and not as a standalone diagnostic tool.

#### 3.11.2 Syndecan-1

The marker of glycocalyx degradation, syndecan-1, is elevated early in sepsis and is correlated with endothelial damage and microvascular injury. It is an early marker for disease progression into shock and may help in early risk stratification (Charles and Gibot, 2014). Puskarich et al. (2016) reported an AUROC of 0.58 for syndecan-1 measured at emergency department enrolment during protocolised resuscitation for severe sepsis/septic shock in predicting the need for endotracheal intubation, with sensitivity 46% and specificity 73% at a cut-off of 240 ng/mL.

### 3.12 Coagulation and sepsis-induced shock

Abnormalities of coagulation are common in sepsis and lead to the formation of microvascular thrombosis, organ failure and shock. Important markers of coagulation, D-dimers and proteins C, are helpful in clarifying sepsis pathophysiology and prognosis.

#### 3.12.1 Study design and setting (ED/ward/ICU; prospective vs. retrospective)

All evidence related to coagulation and fibrinolysis biomarkers in shock-relevant sepsis is based on intensive care unit (ICU)-based observational studies, which can include retrospective registries as well as single-center prospective cohorts. A big multicentre retrospective study, i.e., the Japanese nationwide registry of patients with sepsis-induced coagulopathy and receiving recombinant human soluble thrombomodulin produced a sepsis-induced coagulopathy (SIC) score by applying routine coagulation variables taken just before the commencement of treatment in emergency and critical care facilities (Iba et al., 2017). An adult ICU observational study of the future viability, predetermined by thoroughly evaluating a diverse hemostatic panel, including global tests, thrombin-generation measurements, anticoagulant-pathway, and fibrinolysis-markers at ICU admission in plasma samples, was aimed to predict the onset of overt disseminated intravascular coagulation (DIC) and short-term outcomes (Koyama et al., 2014). Within the setting of septic shock in particular, a prospective monocentre cohort is used to measure ADAMTS13 parameters along with von Willebrand factor (vWF) and interleukin -6 (IL -6) at baseline to determine mortality risk in the absence of assigning a patient to the open category of DIC (Peigne et al., 2013).

### 3.13 Population characteristics (adult vs. pediatric; severity spectrum; inclusion criteria)

All evidence related to coagulation and fibrinolysis biomarkers in shock-relevant sepsis is based on intensive care unit (ICU)-based observational studies, which can include retrospective registries as well as single-center prospective cohorts. A big multicentre retrospective study, i.e., the Japanese nationwide registry of patients with sepsis-induced coagulopathy and receiving recombinant human soluble thrombomodulin produced a sepsis-induced coagulopathy (SIC) score by applying routine coagulation variables taken just before the commencement of treatment in emergency and critical care facilities (Iba et al., 2017). An adult ICU observational study of the future viability, predetermined by thoroughly evaluating a diverse hemostatic panel, including global tests, thrombin-generation measurements, anticoagulant-pathway, and fibrinolysis-markers at ICU admission in plasma samples, was aimed to predict the onset of overt disseminated intravascular coagulation (DIC) and short-term outcomes (Koyama et al., 2014). Within the setting of septic shock in particular, a prospective monocentre cohort is used to measure ADAMTS13 parameters along with von Willebrand factor (vWF) and interleukin −6 (IL -6) at baseline to determine mortality risk in the absence of assigning a patient to the open category of DIC (Peigne et al., 2013).

### 3.14 Sampling window and kinetics-relevant timing (recognition; 0–3 h, 3–6 h, 6–24 h; single vs. serial)

Timing is more of a standard in coagulation researches in comparison to cytokine analyses; however heterogeneity remains with regard to the time of the index, which can be described as ICU admission and inclusion, or just before therapy. SIC analysis used platelet count, PT ratio, fibrin-degradation products (FDP), and SOFA score at the moment right before the beginning of anticoagulant therapy, which has successfully tied sampling into a moment of decision-making during treatment, unlike emergency department recognition (Iba et al., 2017). The prospective ICU study used a single admission sample (baseline at ICU entry) to predict overt DIC within the first 5 days and 28 days mortality, illustrating that early measurement of thrombin-antithrombin complexes (TAT), plasminogen activator-inhibitor-1 (PAI-1) and protein C can identify patients with severe, ongoing coagulopathy at the onset of sepsis (Koyama et al., 2014). The septic-shock group had ADAMTS13/vWF measured at inclusion (early shock window) and thus this confirmed the notion that, endothelial-linked hemostatic dysregulation exists early on in shock which may not necessarily correlate with DIC scores (Peigne et al., 2013).

### 3.15 Comparator/reference standard (clinical criteria, Sepsis-3 shock definition, lactate, SOFA/qSOFA, DIC/SIC tools)

For coagulation-related biomarkers, reference standards often include (a) clinical severity/organ dysfunction scales (e.g., SOFA), (b) disseminated intravascular coagulation (DIC) scoring systems, and (c) shock definitions where relevant. For example, studies evaluating sepsis-induced coagulopathy (SIC) compare routine coagulation variables and SOFA against established DIC scores such as JAAM-DIC in relation to mortality (Iba et al., 2017). When operationalizing overt DIC, the ISTH overt DIC framework is commonly used (Taylor et al., 2001), and ISTH SSC communications summarize the rationale for SIC as an earlier-stage construct and its relationship to overt DIC (Iba et al., 2023). When septic shock comparators are required, Sepsis-3 criteria are typically used (vasopressor requirement to maintain MAP ≥65 mmHg with lactate >2 mmol/L in the absence of hypovolemia) (Singer et al., 2016; Shankar-Hari et al., 2016).

### 3.16 Endpoints and prediction horizon (transition to vasopressor-dependent shock; time-to-shock; mortality)

Endpoints in coagulation studies are usually mortality and/or evolving to overt DIC, but not prospective time-to-vasopressor shock. The 28-day mortality was the main outcome of the SIC study; SIC score positivity (≥4) was suggested as the predictor of increased mortality and offered to the choice of participants to use anticoagulant (Iba et al., 2017). The study was an ICU prospective biomarker to examine overt DIC within a period of 5 days and 28 days, TAT/PAI-1/Protein C showed great discrimination in overt DIC within 5 days of the study, and TAT/PAI-1 was a predictor of mortality (Koyama et al., 2014). ADAMTS13 activity below 30% independently predicted hospital mortality in the condition of septic shock, indicating that it has prognostic value even in the absence of DIC conditions (Peigne et al., 2013).

### 3.17 Validation approach (internal vs. external; calibration/transportability)

There is still unequal external validation on definitive coagulation biomarkers. The derivation of the SIC score used a big multicentre dataset, and therefore it was more representative; nevertheless, it remained just a single-country registry anchored to the treated subgroup (rhTM recipients), which is why it needed to be confirmed that it could be transported to other ED recognition cohorts (Iba et al., 2017). The intent to develop ICU biomarker (TAT/PAI-1/Protein C) was single center and hypothesis generating, it helps internal discrimination but requires replication across sites (Koyama et al., 2014). The ADAMTS13 septic-shock cohort was also monocentric, which promotes biological plausibility and prognostic signal, yet still demands multi-cohort validation and calibration reporting prior to bedside use (Peigne et al., 2013).

#### 3.17.1 D-dimers

High levels of D-dimer represent enhanced fibrinolysis and are highly related to disseminated intravascular coagulation (DIC), a complication of sepsis. The use of d-dimer measurement is helpful to evaluate the condition of both the severity of sepsis and mortality (Nesaragi and Patidar, 2020).

In emergency department cohorts, higher D-dimer levels are associated with worse outcomes and predict short-term mortality in patients with suspected infection or sepsis (Rodelo et al., 2012). D-dimer has also been evaluated alongside procalcitonin for outcome risk assessment and severity stratification in sepsis populations, although performance varies by cohort and endpoint definition (Naderpour et al., 2019).

#### 3.17.2 Protein C

Low levels of protein C, an important anticoagulant, are markers of endothelial dysfunction and hypercoagulability. Lowered levels of protein C are associated with a worse outcome in sepsis, including an increased risk for organ failure and death (Wang et al., 2023). Koyama et al. (2014) reported an AUROC of 0.85 for protein C activity measured within 6 h of ICU admission in predicting progression to overt DIC within 5 days in sepsis patients, with 81% sensitivity and 79% specificity at a cut-off of 46%.

## 4 Immune dysregulation biomarkers

Sepsis is increasingly considered a biphasic disorder with the initially hyperinflammatory phase followed by the phase of immunosuppression. This immunovariation has a central role in the pathogenesis of sepsis-induced shock, precipitating damage to vital organs, adverse outcomes for patients, and an increased incidence of secondary infections. In sepsis, the mechanisms of the immune dysfunction are not limited to the immune cell exhaustion but include immunocell function-altering, apoptosis, and immune responses-dysregulation. The present section considers the most important biomarkers related to the immune dysregulation, such as monocyte and lymphocyte dysfunction, neutrophil activation, immune cell markers, which decipher complex immune response in sepsis, and provide a possibility for targeted immunomodulatory therapies (Wu et al., 2024).

### 4.1 Monocyte and lymphocyte dysfunction

The dysfunction of antigen-presenting cells, specifically monocytes, and the subsequent apoptosis of lymphocytes is a hallmark of sepsis. Such immunological alterations have been linked to immune paralysis and are predictive of adverse outcomes such as increased vulnerability to secondary infections, multi-organ failure and death. Monitoring these immune cell dysfunctions can be invaluable in elucidating a patient’s immune status and ability to respond to infection (Singer, 2020).

Monocyte and Lymphocyte Dysfunction (mHLA-DR; Stimulated Whole-Blood Functional Assays; Lymphopenia) — Structured Synthesis Using a Uniform Evidence Rubric.

### 4.2 Study design and setting (ED/ward/ICU; prospective vs. retrospective)

Standardized flow-cytometric measurement of monocytic human leukocyte antigen-DR (mHLA-DR) is evidence that monocyte dysfunction occurs mostly due to observational cohorts that are conducted in intensive care units (ICUs). A cohort study was implemented in one ICU in the screening of patients with septic shock, and linear plots of serial mHLA-DR were performed in the first week of illness (Leijte et al., 2020). An expanded, clinical-world group of ICU patients in 2005–2024 reports of routine mHLA-DR measurement of septic shock and relates these values to clinical outcomes (Monneret et al., 2025). Further ICU evidence is based on observational cohort of surgical ICU that compared early and late mHLA-DR with the ICU-acquired infections (de Roquetaillade et al., 2022).

To achieve viable immune studies, *ex vivo* whole-blood cytokine secretion on lipopolysaccharide (LPS) stimulation is commonly regarded as a marker of immune suppression of critical illness yet the variable data produced by methodology requires standardisation (Segre and Fullerton, 2016). The recent outcome of multicentre work introduced a whole-blood ELISpot assay (TNF/IFNgamma) which endotypes the patients in septic and critically ill states and matched such endotypes with clinical outcomes (Price et al., 2024).

### 4.3 Population characteristics (adult vs. pediatric; severity spectrum; inclusion criteria)

Most immune dysfunction datasets are derived from adult, high-severity ICU cohorts and are often restricted to septic shock. In one serial-trajectory cohort, patients were enrolled as septic shock and (for enrollments prior to 2016) retrospectively harmonized to Sepsis-3 shock criteria (vasopressor requirement with lactate >2 mmol/L) (Leijte et al., 2020). A longitudinal ICU cohort similarly focuses on septic shock with repeated immune monitoring over time (Monneret et al., 2025). Hematologic immune impairment is also captured using readily available indices; for example, persistent lymphopenia has been evaluated as a prognostic factor in adults with bacteremia and sepsis (Drewry et al., 2014).

### 4.4 Sampling window and kinetics-relevant timing (recognition; 0–3 h, 3–6 h, 6–24 h; single vs. serial)

The biomarkers of immune dysfunction are inherently kinetic. Serial mHLA-DR has been measured at multiple early ICU time points in septic shock, including protocols that quantify mHLA-DR on days 1–2, 3–4, and 6–8 to enable trajectory clustering (early improvers vs. delayed/non-improvers vs. decliners) (Leijte et al., 2020). In another ICU cohort, mHLA-DR was measured during the first 3 days after ICU admission, with a second measurement between days 5–10 to distinguish early depth of suppression from persistent low expression (de Roquetaillade et al., 2022). For lymphocyte dysfunction, the day-4 absolute lymphocyte count (persistent lymphopenia) has been associated with outcomes after sepsis diagnosis (Drewry et al., 2014). Functional assays are also time-sensitive and method dependent: ELISpot endotyping studies have assessed LPS-stimulated cytokine responses across the first 7 ICU days (Price et al., 2024), and methodological work highlights that results are sensitive to pre-analytic and analytic conditions (Segre and Fullerton, 2016).

### 4.5 Comparator/reference standard (clinical criteria, Sepsis-3 shock definition; lactate; SOFA/qSOFA; other tools)

Across immune dysfunction studies, the reference standard is typically a clinical septic shock definition (often aligned to Sepsis-3 constructs) and/or severity scores (SOFA/SAPS/APACHE), rather than an alternative biomarker. In the serial ICU trajectory cohort, septic shock enrollment was retrospectively harmonized to Sepsis-3 criteria (Leijte et al., 2020). For lymphopenia, comparators commonly contrast persistent vs. resolving lymphopenia (i.e., changes in absolute lymphocyte count) over the first 4 days (Drewry et al., 2014).

### 4.6 Endpoints and prediction horizon (transition to vasopressor-dependent shock; time-to-shock; mortality as secondary)

These biomarkers are most commonly associated with unfavorable outcomes following the onset of shock and not the occurrence of shock. Secondary infections and mortality over the ICU/hospital horizons are often chosen as endpoints in the ICU cohort of publiced sepsis-associated studies, especially in septic shock ICU cohorts. The effect of delayed/non -improving or decreasing mHLA -DR patterns indicates a greater risk of a composite adverse outcome (secondary infection or death) than does early improver (Trajectory work) (Leijte et al., 2020). The mHLA -DR is also correlated with mortality and ICU-acquired infections over a long observation window (Monneret et al., 2025) as well as with long-term ICU cohorts linking persistently low mHLA -DR with ICU-acquired infections (de Roquetaillade et al., 2022). In dysfunction of lymphocytes, day-4 lymphopenia is an indicator of 28-day mortality and prognosis in the long term (Drewry et al., 2014). Whole-blood ELISpot is a type of functional endotyping that establishes a connection between immune-response endotypes and discharge disposition, in-hospital mortality, as well as longer-horizon mortality (e.g., 180-day) (Price et al., 2024).

### 4.7 Validation approach (internal vs. external; calibration/transport-ability)

With the exception of a few single-center mHLA-DR cohorts, most studies validating mHLA-DR internally (internal validation/association) still have large samples but follow-up is often long-term (Monneret et al., 2025). Multicentre observational designs (with greater transport-ability) have become the new functional endotyping approach, though it only needs more extensive replication and direct calibration/clinical utility testing before bedside decision rules can be relied on (Price et al., 2024). The cross-study comparability of stimulated whole-blood cytokine assays requires methodological standardization which is a major condition to cross-study comparison.

#### 4.7.1 Human leukocyte Antigen-DR (HLA-DR)

HLA-DR is an important molecule on the surfaces of monocytes and macrophages whose primary role involves antigen presentation. In sepsis, HLA-DR expression decreases (monocyte deactivation), reducing effective antigen presentation and contributing to immunoparalysis, secondary infections, and worse outcomes. Serial monitoring of mHLA-DR can therefore track evolving immune suppression (Radygina and Mochalova, 2023). In ICU day-1 cohorts, mHLA-DR showed strong discrimination between sepsis and healthy controls (AUROC 0.91; sensitivity 83.52% and specificity 82.02% at a cut-off of ∼30% HLA-DR) (Wang et al., 2018); however, because sampling occurs after ICU admission and the endpoint is sepsis diagnosis rather than ED-timepoint shock escalation, we treat this as diagnostic/biological plausibility evidence rather than evidence for early shock prediction at presentation.

#### 4.7.2 Lymphopenia and Interleukin-10 (IL-10)

Sepsis is also characterized by extreme apoptosis of T cells, natural killer (NK) cells, and B cells, causing lymphopenia. This acute lymphocyte deficit exacerbates further the immune system’s inability to combat infections and contributes to tissue replacement. In addition, increased IL-10, the anti-inflammatory cytokine, is another hallmark of the immune system. To handle the Immune system, IL-10 is essential for promoting immune tolerance and inhibiting inflammatory action. Persistent increased IL-10 levels in sepsis indicate continuous immune suppression, a predictor of failure to eradicate pathogens, and an increased risk of secondary infections. High levels of IL-10 are common in patients with dire prognoses, which calls for therapeutic approaches that control the immune response (Samraj et al., 2013).

In combination, the mis-regulated immune response of monocytes and lymphocytes is of fundamental importance in this clinical setting, not only to define immunosuppressive sepsis, but to explain why this is the final diagnosis, in circumstances where the initial state is hyper-inflammatory.

#### 4.7.3 Endotoxin tolerance and monocyte deactivation

Endotoxin tolerance represents a central paradigm of the sepsis-related immune suppression; it represents a mechanism in which the prior exposure to microbial elements like lipopolysaccharide (LPS) provokes a reduced innate cytokine response (Hotchkiss et al., 2013). This phenomenon might be clinically manifested by the sustained apathy of the monocytes, failure in antigen presentation, and increased vulnerability to secondary pathogen attack-of which other factors play a crucial role in human deterioration following initial resuscitation. Reduced monocyte HLA-DR (mHLA-DR) expression in this conceptual framework is viewed not only as a fixed prognostic marker but rather as an enhanced biomarker of a biologically plausible immunosuppressed phenotype (Cavaillon and Adib-Conquy, 2006).

#### 4.7.4 Functional immune assays (LPS-stimulated TNF-α release)

Other forms of surface phenotyping include functional assays that measure immune responsiveness, such as *ex vivo* TNF-α release following LPS exposure, to assess innate cytokine-producing capacity (Heagy et al., 2000). These assays may complement mHLA-DR trajectories by distinguishing transient downregulation from persistent immune paralysis, thereby refining risk stratification for secondary infection and late hemodynamic deterioration (primarily in the 6–24 h monitoring decision window and beyond) (Wheelwright et al., 2023). However, clinical use remains limited by assay standardization, turnaround time, and required laboratory infrastructure.

### 4.8 Measurement considerations

To reach the clinical utility associated with mHLA-DR, reporting needs to be defined to include assay platform (flow cytometry), gating strategy and controls, need to have standard units of expression and inter-laboratory harmonization processes. Pre-analytical and cytometric variability can introduce a variation of threshold values of significant scope.

### 4.9 Neutrophil activation and dysfunction

Neutrophils are important parts of the innate immune response and are usually the first responders to an infection. In sepsis, their dysregulated activation and prolonged survival lead to tissue damage, organ damage, and, subsequently, shock. The neutrophils are involved in phagocytosis, and they give reactive oxygen species (ROS) and neutrophil extracellular traps (NETs) that might worsen tissue damage (Walley, 2013).

### 4.10 Uniform evidence-extraction framework across biomarker classes

To achieve a standardized synthesis across biomarker categories, we extracted a common set of study-level variables: (1) study design and setting (ED/ward/ICU; prospective vs. retrospective); (2) population characteristics (adult vs. pediatric; severity spectrum; inclusion criteria); (3) sampling window and timing relative to kinetics (e.g., at recognition, 0–3 h, 3–6 h, 6–24 h; single vs. serial sampling); (4) comparator/reference standard (Sepsis-3 septic shock definition, lactate/SOFA, clinical adjudication, or other prespecified criteria); (5) endpoint and prediction horizon; and (6) model/performance reporting (AUROC, sensitivity, specificity, and calibration when available). Performance metrics derived from diagnostic comparisons (e.g., sepsis vs. controls) are explicitly labeled as diagnostic discrimination and are not treated as evidence of early shock prediction at presentation unless the endpoint is shock/vasopressor initiation within a defined horizon.

### 4.11 Study design and setting (ED/ward/ICU; prospective vs. retrospective)

Evidence comes from ED triage/progression cohorts as well as ICU prognostic cohorts. Heparin-binding protein (HBP), a neutrophil-derived mediator of vascular leak, has been evaluated as an early warning marker for organ dysfunction in suspected infection in a large prospective international multicentre ED cohort (Linder et al., 2015). A separate prospective hospital cohort assessed HBP for sepsis and septic shock discrimination (Zhou et al., 2019). Neutrophil activation has also been studied using neutrophil CD64 (nCD64) upregulation, with serial measurements at ICU admission and 48 h later to refine prognostic stratification (Pham et al., 2023). Circulating neutrophil extracellular trap (NET) remnants (e.g., MPO–DNA complexes and cell-free DNA) have been quantified by serial sampling after shock onset in multicentre ICU cohorts (Maruchi et al., 2018). Calprotectin (S100A8/A9) is another neutrophil/monocyte-derived biomarker evaluated in prospective ICU cohorts for sepsis discrimination and mortality risk (Larsson et al., 2020).

### 4.12 Population characteristics (adult vs. pediatric; severity spectrum; inclusion criteria)

The demographics of the populations (adult vs. pediatric; spectrum of severity; inclusion criteria) demonstrate that the majority of the cohorts use populations of adults that have an enriched high-acuity missing. The ED HBP study was done among adults who reported to the hospital with the suspicion of being infected and showing signs of systemic inflammatory response, and followed the event of organ diseases development within 72 h (Linder et al., 2015). The Sepsis-3 aligned HBP stratified adults in the healthy, local infection, and sepsis (non-shock) and septic shock (Zhou et al., 2019). The ICU nCD64 cohort included new adult patients with matched Sepsis-3, but it is clear that sepsis and septic shock are not mixed in the cohort upon admission (Pham et al., 2023). The NET -remnant group included ICU patients with septic shock at several centres in Japan (Maruchi et al., 2018). The calprotectin ICU study included all ICU admissions within more than a year, and compared sepsis and non-sepsis conditions and simultaneously considered 30 days mortality (Larsson et al., 2020).

### 4.13 Sampling window and kinetics-relevant timing (recognition; 0–3 h, 3–6 h, 6–24 h; single vs. serial)

Biomarkers were measured on admission to the ED and 12–24 h later in the ED HBP cohort, biomarkers tended to increase in HBP, and at at least 10.5 h before organ dysfunction in deteriorating patients (Linder et al., 2015). HBP concentrations at enrolment were used to diagnose discrimination in Sepsis-3 HBP cohort (Zhou et al., 2019). In the ICU nCD64 study, nCD64 data were recorded at time T0 (admission) and T48 (48 h) producing a kinetics-sensitive trend measure, denoted as percent change in nCD64 (percent change). In the NET−remnant cohort, the initial blood sample was collected within 6 h of fulfillment of the septic shock dimension, followed by those on day 1, 3 and 7: this design showed that prognostic indicators of MPO–DNA might become more significant at later early times 7 (days 37) compared with day 1 (Maruchi et al., 2018). In the calprotectin cohort, a single timepoint of early ICU admission plasma was collected and evaluated regarding their sepsis discrimination and 30-day mortality (Larsson et al., 2020).

### 4.14 Comparator/reference standard (Sepsis-3 shock definition; lactate; SOFA/qSOFA; other tools)

Reference standards and comparators (Sepsis-3 septic shock definition; lactate; SOFA/qSOFA; other tools) vary across studies, although most are anchored to Sepsis-3 constructs. For example, an HBP cohort used Sepsis-3 criteria to categorize sepsis and septic shock and compared HBP performance with SOFA and conventional inflammatory biomarkers (Zhou et al., 2019). An ICU nCD64 cohort likewise applied Sepsis-3 septic shock criteria (vasopressor requirement with lactate thresholds) and compared nCD64-based measures with SOFA and APACHE II (Pham et al., 2023). In an ED progression cohort, infection and organ dysfunction during the first 72 h were used as reference outcomes and compared across HBP, lactate, procalcitonin, and C-reactive protein (Linder et al., 2015). NET remnant studies validated septic shock in ICU cohorts and related MPO–DNA complexes to physiologic and organ dysfunction markers (e.g., MAP, P/F ratio, SOFA) (Maruchi et al., 2018). Calprotectin studies have compared sepsis discrimination and 30-day mortality prediction against procalcitonin (Larsson et al., 2020).

### 4.15 Endpoints and prediction horizon (transition to vasopressor-dependent shock; time-to-shock; mortality)

The ED HBP investigation targeted organ dysfunction or severe sepsis development during the first 72 h, which justifies its position as an early sign of deterioration (Linder et al., 2015). The cohort (Sepsis-3 HBP) concentrated on the accuracy of diagnosis at presentation/enrolment and did not find a notable difference in the enrolment HBP levels between 28-day survivors and non-survivors (Zhou et al., 2019). In the case of nCD64, the ICU cohort found better predictive ability using higher reaches of nCD64 and 48-h trend of nCD64-percent change (IC) showed a better prognosis (Pham et al., 2023). In NET biology, the MPO–DNA levels were related to the degree of dysfunction of the organs and correlated with the mortality, 28 days later (at days 3 and 7), in the measured timepoint (Maruchi et al., 2018). At the ICU admission, calprotectin had a better discriminatory performance concerning sepsis, and had a higher predictive value concerning 30-day mortality compared to procalcitonin in that cohort of ICU patients (Larsson et al., 2020).

### 4.16 Validation approach (internal vs. external; calibration/transportability)

The ED HBP cohort included an institutional external validation group from a second centre, improving transportability compared with single-centre analyses (Linder et al., 2015). In contrast, the Sepsis-3 HBP cohort was a single-hospital study, highlighting the need for broader external validation in diverse settings and case mixes (Zhou et al., 2019). The ICU nCD64 cohort was also single-centre, indicating a need to replicate kinetics-based nCD64 measures across multiple sites (Pham et al., 2023). The NET remnant cohort used a multicentre ICU design but did not provide an independent external validation cohort for predictive modelling (Maruchi et al., 2018). Calprotectin evidence is likewise largely derived from single-ICU cohorts and requires cross-site validation before routine clinical adoption (Larsson et al., 2020).

#### 4.16.1 CD64 expression on neutrophils (FcγRI)

CD64, which has also been identified as the high-affinity receptor for the Fc portion of IgG, is only expressed in the resting neutrophils in minimal amounts, but is upregulated in the next 4–6 h of exposure to bacterial components or inflam High level expression of CD64 is an early sensitive and specific biomarker of bacterial infection and sepsis. Its relationship was found with systemic inflammation, disease severity and risk of shock. Unlike other markers such as CRP, CD64 can help discriminate infectious from non-infectious inflammation triggers, a practical aspect in early diagnosis. Furthermore, CD64 expression on neutrophils has the potential to be a guide for antimicrobial therapy, assisting clinicians in deciding to start or de-escalate antimicrobial therapy (Cioara et al., 2016).

#### 4.16.2 Neutrophil extracellular traps (NETs)

NETs, part of the innate response, are web-like structures formed from DNA and antimicrobial proteins released by neutrophils when infected. The role of NETs is homing in pathogens, and their uncontrolled formation in sepsis is accompanied by damage to tissues, microvascular thrombosis and dysfunction of the organs. High levels of NETs have been linked to a bad outcome in septic patients, especially in those who develop into septic shock. New research is concerned with targeting NET formation as a therapeutic avenue to minimize the collateral damage from the activation of neutrophils (von Groote and Meersch-Dini, 2022).

#### 4.16.3 Myeloperoxidase (MPO)

MPO is an enzyme stored in the neutrophils, which takes part in the reactive oxygen species (ROS) when confronting the immune response. High levels of MPO in sepsis reflect activated neutrophils and oxidative stress, both underlying factors of tissue damage and organ failure. MPO is related to the disease severity and has potential as a marker predicting septic shock and mortality risk (Venter et al., 2010).

Therefore, neutrophil dysfunction and activation markers, including CD64, MPO, and NETs, provide helpful information regarding the pathogenesis of sepsis-induced shock, and monitoring such will contribute to the early diagnosis and correctional procedures.

### 4.17 Immune cell markers and new insights

Besides HLA-DR, IL-10, CD64 and NETs, a range of new immune cell markers and genomic signatures are gaining prominence as potent markers of early diagnosis of sepsis and monitoring of immune status. Markers are essential for determining patients at risk of developing immunosuppression and patients who can gain from immunomodulatory therapy (Fraunberger and Walli, 2007).

### 4.18 Study design and setting (ED/ward/ICU; prospective vs. retrospective)

Evidence for immune cell-state biomarkers in sepsis-induced shock largely comes from observational ICU and ED cohorts that assess surface and soluble checkpoint/exhaustion markers as well as immune function-related transcripts. In one ED-based cohort, monocyte PD-L1 measured in the late immunosuppressive phase (days 3–4 after sepsis onset) correlated with severity and mortality (Shao et al., 2016). A smaller ICU septic shock cohort performed serial immunophenotyping of CD4^+^ T cells (days 1, 3, and 7) alongside T-cell receptor (TCR) repertoire diversity to capture dynamic immune-state changes (Tomino et al., 2017). Complementary transcript markers such as CD74 and IL7R have been profiled around day 3 after shock diagnosis in ICU cohorts and linked to prognostic enrichment (Cazalis et al., 2013; Delwarde et al., 2018).

### 4.19 Population characteristics (adult vs. pediatric; severity spectrum; inclusion criteria)

Population characteristics vary across cohorts with respect to age, baseline severity, and inclusion criteria. Most studies in this subsection are adult cohorts enriched for higher-severity presentations (sepsis or septic shock). The ED PD-L1 study included distinct sepsis and septic shock groups and focused on later sampling (days 3–4) to capture the immunosuppressive phase (Shao et al., 2016). ICU investigations of PD-1 expression and TCR diversity typically involve repeated sampling over the first week after shock onset (Tomino et al., 2017). Transcript-based biomarkers have likewise been studied primarily in adult ICU septic shock cohorts, including day-3 assessments of CD74 (Cazalis et al., 2013).

### 4.20 Sampling window and kinetics-relevant timing (recognition; 0–3 h, 3–6 h, 6–24 h; single vs. serial)

Sampling windows and timing are crucial for interpreting immune cell markers. Checkpoint/exhaustion markers can be informative during early resuscitation (within the first 24–48 h) and during the subacute period of immunosuppression. For example, monocyte PD-L1 was measured on days 3–4 after sepsis onset in an ED cohort (Shao et al., 2016). Serial sampling improves interpretability: PD-1 expression and TCR diversity assessed on days 1, 3, and 7 reflected ongoing immune dysfunction in non-survivors (Tomino et al., 2017). Transcript markers such as CD74 and IL7R mRNA are typically measured around day 3 after shock recognition (Cazalis et al., 2013; Delwarde et al., 2018). For soluble inhibitory receptors, sBTLA measured within the first 24 h of ICU admission illustrates an early critical illness window (Lange et al., 2017).

### 4.21 Comparator/reference standard (clinical criteria; Sepsis-3 shock definition; lactate; SOFA/qSOFA; other tools)

Studies typically benchmark biomarkers against standard clinical criteria—such as Sepsis-3 shock definitions, lactate, SOFA and qSOFA scores, and other physiologic measures—which serve as comparators/reference standards. ED studies (e.g., PD-L1) have tested incremental value versus traditional clinical stratification (sepsis vs. septic shock) and established risk scores (Shao et al., 2016). In ICU settings, studies often compare biomarker performance with SOFA and APACHE II scores; for example, soluble sBTLA associated with SOFA and was assessed for incremental prognostic value (Lange et al., 2017). Similarly, PD-1+CD3^+^ T cells were evaluated in combination with SOFA/APACHE II to test additive value (Wang et al., 2025).

### 4.22 Endpoints and prediction horizon (transition to vasopressor-dependent shock; time-to-shock; mortality; secondary infection)

Across these studies, endpoints and prediction horizons are often mortality or secondary infection risk rather than time to vasopressor-dependent shock. Monocyte PD-L1 measured on days 3–4 has been associated with severity and 28-day mortality (Shao et al., 2016). PD-1 expression and TCR diversity tracked during the first week have also been linked to mortality in septic shock cohorts (Tomino et al., 2017). Day-3 CD74 mRNA has been reported as a predictor of 28-day mortality after septic shock (Cazalis et al., 2013), and IL7R mRNA measured around day 3 has shown prognostic value with validation suggesting potential rule-out utility (Delwarde et al., 2018). In downstream complications, combined CD74/IL10 mRNA dynamics have been associated with ICU-acquired infections, consistent with immune suppression biology (Peronnet et al., 2017).

### 4.23 Validation approach (internal vs. external; calibration/transportability)

Internal and external validation strategies remain heterogeneous and are a major barrier to bedside translation. Single-centre designs and small cohorts limit transportability (Shao et al., 2016; Tomino et al., 2017). Stronger examples include IL7R mRNA assessed in a discovery cohort and confirmed in an independent multi-ICU validation cohort, demonstrating cross-site robustness (Delwarde et al., 2018). A multicentre study of ICU-acquired infection linked CD74 and IL10 mRNA dynamics to that endpoint, improving generalizability for infection risk (though not necessarily mortality prediction) (Peronnet et al., 2017). Recent studies of PD-1+CD3^+^ T cells often rely on internal bootstrap validation and still require external cohorts to establish transportability (Wang et al., 2025).

#### 4.23.1 Immune cell infiltration and the gene signatures

The recent study has concentrated on the gene expression profiles that define the phenomenon of immune cell infiltration and immune response in sepsis. Specific gene signatures that quantify the amount of immune dysregulation in sepsis have been attained using bioinformatics techniques. For example, the CD177/MMP8 ratio has promising prospects for discerning bacterial infection from viral infection and predicting the development of septic shock. Also, attracting attention is the CXCR3/CCR5 axis, which is dysregulated at the initial stage of sepsis, and can become a promising therapeutic target for early therapy (Limongi et al., 2016).

#### 4.23.2 T-cell immunoregulatory markers

Markers including programmed cell death protein 1 (PD-1), CTLA-4 are increased in sepsis, further leading to T cell exhaustion and immune suppression. These markers are being investigated as probable targets for immunotherapy in sepsis and, through the correction of immune function, enhance patient outcomes (Ivady et al., 2011).

Immune dysregulation in sepsis is the key underlying factor in mortality from the disease to septic shock. Measurement of important biomarkers of monocyte dysfunction, lymphocyte apoptosis, neutrophil activation, and immune cell infiltration gives critical information on the immune status of septic patients. In addition to being indicators of the degree of immune dysfunction, these biomarkers provide possible routes of immunomodulatory intervention. Early identification of immune suppression may be utilized to inform the use of immunotherapies to restore immune function and improve patient outcomes in sepsis-induced shock (Sсherbak et al., 2023).

A compilation of the significant studies used to assess biomarkers for sepsis-induced shock is shown in Table 4.

**TABLE 4:** Summary of key studies on biomarkers for sepsis-induced shock, including methods, findings, and clinical implications.

Study	Insights	Methods used	Practical implications	Findings/conclusions	Representative quantitative metrics (where reported)
Tanak et al. (2022)	DETecT multi-biomarker device predicts sepsis outcomes by measuring seven circulating markers and integrating them into a composite score	Point-of-care multi-analyte device with embedded machine-learning model; comparison with reference laboratory assays	Supports rapid ED risk-stratification and personalized sepsis management at the bedside	Strong correlations between device and reference assays; composite score predicted sepsis severity and outcome with high accuracy	Internal validation reported good discrimination with AUROC values in the high 0.8–0.9 range for sepsis and short-term adverse outcomes; exact per-marker AUROCs vary by cohort and are not central to this review
Li et al. (2021) (clinical biomarkers)	IL-6, NT-proBNP and INR emerge as key biomarkers for predicting 28-day mortality in sepsis	Prospective sepsis cohort; serial biomarker measurement; comparison with SOFA and APACHE II scores	Combining inflammatory and cardiac markers can refine early mortality prediction and guide escalation of care	IL-6, NT-proBNP and INR together outperformed SOFA and APACHE II for 28-day mortality prediction	Reported AUROC values for combined biomarker models generally ≥0.80 for 28-day mortality, higher than SOFA/APACHE II alone; NT-proBNP cut-offs around 2,000–4,000 pg/mL showed the best trade-off between sensitivity and specificity
Wang et al. (2023)	High-throughput proteomics and transcriptomics identify molecular signatures associated with sepsis-induced shock	Omics profiling (mass spectrometry and RNA-seq), pathway analysis, and network modelling	Provides candidate biomarker panels and pathways for early diagnosis and prognosis of septic shock	Multi-protein and gene signatures showed clear separation between septic shock, sepsis and controls	Discovery-phase ROC analyses reported high diagnostic AUCs (often ≥0.85) for multi-marker panels; however, estimates are exploratory and require external validation
Chaithanya and Meshram (2023) (meta-analysis)	Cytokines, chemokines and acute-phase proteins act as early indicators of sepsis and mortality risk	Systematic review and meta-analysis of biomarker studies in adult sepsis	Helps prioritise biomarkers (e.g., IL-6, PCT, sTREM-1) that are most promising for integration into clinical algorithms	Biomarkers such as IL-6 and sTREM-1 consistently showed better performance than CRP alone for mortality prediction, but heterogeneity across studies was substantial	Pooled AUROC values for leading markers typically in the 0.75–0.85 range for sepsis diagnosis and mortality, with combined panels performing better than single markers
He et al. (2024) (emerging biomarkers)	Novel markers including circular RNAs and miR-486-5p may improve sepsis diagnosis and prognosis	Narrative review of emerging molecular biomarkers and their clinical data	Supports future development of multi-omic panels and precision-medicine approaches in sepsis	Many emerging markers show higher reported sensitivity/specificity than traditional biomarkers in small cohorts, but lack large-scale validation	Reported individual studies with AUROC values often >0.80 for select non-coding RNAs; however, evidence is early-stage and based on small single-centre cohorts
Ciriello et al. (2013)	Early sepsis biomarkers include pro-inflammatory cytokines and acute-phase proteins such as IL-6, PCT and lactate	Narrative review of clinical and experimental biomarker studies	Highlights the need for multi-marker strategies and dynamic monitoring rather than reliance on a single test	PCT and CRP show useful but imperfect discrimination; lactate adds independent prognostic value	Largely qualitative synthesis; AUROC values are summarised from primary studies but no new pooled estimates are generated
Trancă et al. (2014)	sTREM-1 and presepsin are promising biomarkers for sepsis diagnosis	Review of candidate sepsis biomarkers and their validation status	Underlines the need for rigorous external validation before routine clinical adoption	Many markers show encouraging early AUROC values but suffer from small sample sizes and spectrum bias	Reports original studies with AUROC commonly in the 0.75–0.88 range for sTREM-1 and presepsin in sepsis vs. non-infectious SIRS, but emphasises variability and lack of standardisation
Anderson and Schmidt (2010)	Synthesises clinical and experimental data on cytokines and acute-phase proteins as predictors of sepsis-induced shock	Narrative review of early biomarker and animal work	Frames rationale for combining biomarkers with clinical scores rather than using isolated markers	PCT and CRP are the most widely studied but lack ideal specificity and sensitivity; multi-marker approaches are recommended	Descriptive overview; quantitative performance is reported indirectly via cited primary studies without unified AUROC estimates
Ke et al. (2023)	Machine-learning models can improve sepsis diagnosis and outcome prediction beyond traditional scores	Review of ML algorithms applied to sepsis (e.g., random forests, deep learning) and their validation	Encourages cautious integration of ML models into clinical workflows with strong emphasis on external validation and calibration	Many models achieve AUROC values ≥0.85 for early sepsis detection or mortality prediction in test datasets but often lack transparent reporting and generalisability	Summarises studies where ML tools reach AUROC 0.85–0.95 for sepsis diagnosis or mortality; however, between-study heterogeneity is high and comparative performance vs. SOFA/APACHE varies
Pierrakos et al. (2020)	Reviews cytokines, chemokines and acute-phase proteins in the context of modern sepsis therapies	Comprehensive review incorporating molecular profiling and big-data approaches	Positions biomarkers as tools to select patients for immunomodulation, precision antibiotics and advanced therapies	Concludes that multi-biomarker panels, rather than single markers, are required to meaningfully guide sepsis treatment	Quantitative data presented from multiple original studies (typical AUROC 0.7–0.85 for individual markers); no new pooled ROC estimates are calculated
Li et al. (2021) (microfluidics)	Microfluidic platforms can shorten sepsis diagnostic turnaround and enable point-of-care biomarker testing	Review of traditional and microfluidic methods for sepsis detection	Supports the development of rapid bedside devices that integrate biomarker measurement and microbial diagnostics	Microfluidic systems match or exceed traditional assays in sensitivity and specificity while dramatically reducing time to result in small validation studies	Individual devices report AUROC values commonly ≥0.80 for sepsis or bacteremia detection, but sample sizes are limited and estimates are not yet generalisable
Chaithanya and Meshram (2023) (gene-expression study)	Identifies six hub genes as early biomarkers for septic shock	Bioinformatic analysis of public gene-expression datasets with ROC validation	Gene signatures may support early identification of patients at risk of septic shock and guide targeted therapies	Six genes showed strong differential expression and association with septic shock	Reported ROC curves for hub genes with AUROC typically >0.85 in derivation cohorts; external validation is still limited

## 5 Emerging and novel biomarkers

Sepsis-induced shock and its unpredictable advance have propelled this paradigm shift to fundamentally search for novel biomarkers for early diagnosis, prognosis prediction, and therapeutic targeting. Conventional biomarkers like cytokines and acute-phase proteins have been prolific in shedding light on sepsis’s inflammation and immune response biomarkers. Notwithstanding their limitations, such as late response and lack of specificity, have led to the development of new patterns of molecular signatures from advanced omics technologies (genomics, proteomics, transcriptomics and metabolomics). These new biomarkers represent a collective vision of the host’s response to infection, reflecting immune reprogramming, endothelial dysfunction and metabolic changes, all pivotal in the emergence of sepsis-induced shock (Peng and Liu, 2016).

### 5.1 MicroRNAs (miRNAs) and circular RNAs

MicroRNAs (miRNAs) are short, non-coding RNA molecules known to post-transcriptionally regulate gene expression by binding their sequence to complementary sequences on messenger RNAs (mRNAs). Several biological processes have been identified. Their extreme stability in plasma and degradation resistance make them competitive candidates as sepsis biomarkers (Liu et al., 2014).

Evidence in this subsection was appraised using the uniform evidence-extraction rubric described in Methods (Section 2.4); studies limited to sepsis vs. SIRS/controls are interpreted as diagnostic/biological plausibility evidence rather than ED-timepoint prediction of shock escalation (Tricco et al., 2018; Collins et al., 2015).

### 5.2 Study design and setting (ED/ward/ICU; prospective vs. retrospective)

Most of the evidence is observational, based on critical care and ED-to-ICU pathways, both single-centre in nature and multicentre. Next-generation sequencing (NGS) was combined with quantitative reverse transcription PCR (qRT-PCR) in order to identify if a circulating microRNA distinguishes sepsis and non-infective systemic inflammatory response syndrome (SIRS) in the phenotypically constrained critical illness populations in an ICU admission cohort (Caserta et al., 2016). In the case of sepsis versus septic shock discrimination, multicentric prospective post-operative cohort profiled plasma extracellular vesicle microRNAs (EV-miRNAs) and preselected EV 150 5p as a solid discerning instrument of septic shock, and compared and contrasted to an external database of plasma microRNAs (EVs) (Martín-Fernández et al., 2025). A prospective cohort concept was adopted into the circRNA sector where healthy, pneumonia, and pneumonia-induced sepsis groups were examined through RNA-seq discovery and validation by a qRT-PCR, which was used to examine the diagnostic and prognostic capacity of serum circRNAs (Wang et al., 2025).

### 5.3 Population characteristics (adult vs. pediatric; severity spectrum; inclusion criteria)

The common cohort studies of most “omics RNA biomarkers are in adult cohorts, and high-acuity. The study of ICU miRNA differentiation was focused on the critically ill patients who were classified on admission to the critical care as sepsis, non-infective SIRS, and no-SIRS controls (Caserta et al., 2016). The EV-miRNA clinical trial recruited post-operative patients and the participants were grouped into those developing sepsis and those leading to septic shock (Martín-Fernández et al., 2025). The circRNA article especially tested the sepsis due to pneumonia and analyzed the circRNA concentrations in connection to the severity scores and death (Wang et al., 2025).

### 5.4 Sampling window and kinetics-relevant timing (recognition; 0–3 h, 3–6 h, 6–24 h; single vs. serial)

RNA biomarkers are commonly quantified as a single early sample around ICU/ED identification, despite the kinetic aspects of this being variably reflected by different studies. The ICU miRNA profiling study was a measurement of plasma miRNAs on or before admission to the critical care as the phenotype-defined groups (Caserta et al., 2016). The circRNA cohort measured serum circRNAs between diagnostic groups and used their levels to predict diagnostic ROC as well as 28-day mortality prognostic suggesting a base-level measurement that can be used to predict diagnostic ROC and 28-day mortality prognostic (Wang et al., 2025). Heterogeneity of sampling and normalization is known to be one of the causes of inter-study variability, which was clearly noted in diagnostic meta-analyses in the wider miRNA literature (Shen et al., 2020).

### 5.5 Comparator/reference standard (clinical criteria; Sepsis-3 shock definition; lactate; SOFA/qSOFA; other tools)

#### 5.5.1 Comparators differ

Some of the studies have etiological discrimination (sepsis vs. non-infective SIRS), some have severity (sepsis vs. septic shock), and others are standard clinical scores. Caserta et al. utilized thorough clinical phenotyping as the means of identifying sepsis as opposed to non-infective SIRS among ICU patients (Caserta et al., 2016). The cohort of circRNAs that were correlated with SOFA and the APACHE2 scores measured whether circRNAs can provide diagnostic value over the combination of the common inflammatory markers and severity scores (Wang et al., 2025). Sub-group analysis is proposed to be the best diagnostic accuracy in pooled diagnostic evidence when the standards applied to studies are Sepsis-3 criteria as well as reports performance compared to regular biomarkers like procalcitonin and C-reactive protein (Shen et al., 2020).

### 5.6 Endpoints and prediction horizon (transition to vasopressor-dependent shock; time-to-shock; mortality)

The majority of the RNA-biomarker studies consider classification (sepsis vs. controls/SIRS; sepsis vs. shock) and mortality, but not the actual time-to-shock progression. EV miR-150-5p had an excellent performance to recognize the presence of septic shock and sepsis, and the area under the curve part is reported in the abstract (Martín-Fernández et al., 2025). Both diagnostic separation of sepsis using pneumonia and prognostic anticipation of 28 days death of specific circRNAs were noted in the circRNA cohort (Wang et al., 2025). In the case of circulating miRNA as prognosis, ICU outcomes were related to serum miR-150 levels in a critical illness cohort (including sepsis), specifically to a prognostic (as opposed to diagnostic) effect (Roderburg et al., 2013).

### 5.7 Validation approach (internal vs. external; calibration/transportability)

One of the strengths of the EV-miRNA shock discrimination study is the utilization of a validation cohort and comparison of the external plasma-miRNA dataset (Martín-Fernández et al., 2025). The sepsis induced by circRNA is a prospective study that uses qRT-PCR validation of the RNA-seq results but contains a single-study design and lacks an external sample group to test the transportability (Wang et al., 2025). At the evidence-synthesis level, diagnostic meta-analyses summarize a number of studies (e.g., 22 records; >2,000 patients with sepsis) and highlight explicitly which factors could potentially cause heterogeneity (adult versus pediatric, serum versus plasma, Sepsis-3 use, normalization choices), making it clear that before it can be used at the bedside, harmonized approaches are needed (Shen et al., 2020; Zheng et al., 2023).

#### 5.7.1 miR-150

Downregulation of miR-150 has been detected consistently in patients with sepsis. miR-150 performs a regulatory role in the differentiation and function of the immune cells. Its decreased expression is accompanied by compromised immune function, lymphocyte apoptosis, and poor prognosis. Depressed levels of circulating miR-150 are associated with higher Sequential Organ Failure Assessment (SOFA) scores and high mortality in septic patients.

#### 5.7.2 miR-223

miR-223 is crucial in controlling neutrophil maturation and the resolution of inflammation. Systemic hyperinflammation and susceptibility to shock have been associated with dysregulated miR-223 expression. Because miR-223 can change dynamically with treatment and disease trajectory, it is a candidate marker for real-time sepsis surveillance and risk stratification.

#### 5.7.3 miRNA panels

Previous research has shown the advantage of combinatorial miRNA signatures over individual miRNAs. Such miRNA panels may enhance the predictive precision for the progression of sepsis, the development of shock, and organ dysfunction. The incorporation of these panels into platforms of multiplex PCR may lead to testing-at-point and actionable information about sepsis prognosis (Liu et al., 2014).

#### 5.7.4 Circular RNAs (circRNAs)

Circular RNAs make up one class of non-coding RNAs that are closed covalently and are stable. Recent research revealed the interaction of circRNAs with modulation of immune responses and acting as possible biomarkers of sepsis. For instance, a circRNA, miR-486-5p, has been implicated in the sepsis pathogenesis and may serve as a new diagnostic sepsis marker for early diagnosis (Agnello and Ciaccio, 2023).

### 5.8 Endothelial dysfunction and glycocalyx-associated biomarkers

Endothelial damage is a significant element of septic shock. The endothelial barrier in sepsis is compromised by vascular leakage, hypotension and tissue hypoperfusion. Biomarkers that reflect endothelialization, damage and glycosciolysis are commanding interest as early indicators of sepsis development and shock predisposition (Dolin et al., 2018).

#### 5.8.1 Endocan (ESM-1)

Endocan is an extracellular dermatan sulfate proteoglycan expressed by endothelium upon induction with pro-inflammatory cytokine IL-1 or TNF-α. High levels of endocan are linked to endothelial impairment, heightened vascular permeability, and a grim outcome in septic patients. There is an independent association of high endocan with ICU admission, vasopressor needs and death.

#### 5.8.2 Syndecan-1

Syndecan-1 is one of the endothelial glycocalyx’s associated glycoproteins. In sepsis, the syndecan-1 levels increase early, indicating glycocalyx dissolution and endothelial damage. High syndecan-1 levels are associated with microvascular damage and match the risks of shock, being a probable early biomarker of sepsis aggravation (Dolin et al., 2018).

### 5.9 Transcriptomic and proteomic signatures

Developments in high-throughput sequencing and mass spectrometry-based proteomics have created avenues for the discovery of composite molecular signatures that can be predictive of the progression of sepsis. These signatures merge both immune response, inflammatory trajectories, and metabolic modifications allowing a more rounded understanding of sepsis pathophysiology.

### 5.10 Study design and setting (ED/ward/ICU; prospective vs. retrospective)

The most common findings on the evidence of the signatures of this process show the use of prospective observational intensive care unit (ICU) cohorts using either (i) hostresponse transcript panel which was designed to distinguish between infectious and noninfectious systemic inflammation; (ii) unsupervised wholeblood transcriptomic analyses which identify endotypes of sepsis related to clinical outcomes; (iii) highthroughput plasma proteomics to map modules and subphenotypes of hostresponse. SeptiCyteLab was designed and tested in observational cohorts of observers in adult ICUs, including discovery and independent validation cohorts (McHugh et al., 2015; Miller et al., 2018). Prospective studies of the ICU with endotyping (whole-blood transcriptomic) have been done with multi-ICU validation studies, to correlate the pattern of genomic responses with survival (Davenport et al., 2016; Scicluna et al., 2017). Mass spectrometry (wide) studies have been done on sepsis plasma proteome, providing phenotyping of severity and patterns of organ-failure (Mi et al., 2024).

### 5.11 Population characteristics (adult vs. pediatric; severity spectrum; inclusion criteria)

Shepheard et al. (2017) note that most omics-profile studies are conducted in adult, ICU-enriched sepsis cohorts. Transcriptomic heterogeneity and its relationship to outcomes in ICU patients with community-acquired pneumonia and organ dysfunction has been examined with replication in an independent cohort (Davenport et al., 2016). Genomic endotype classifications have been confirmed across multiple ICU cohorts (e.g., two Dutch ICUs and a UK multi-ICU pneumonia-sepsis cohort), demonstrating endotypes with different mortality risks (Scicluna et al., 2017). Proteomic mapping studies also include broad, unselected sepsis case mixes and comparator groups (Mi et al., 2024).

### 5.12 Sampling window and kinetics-relevant timing (recognition; 0–3 h, 3–6 h, 6–24 h; single vs. serial)

Sampling window and timing (recognition; 0–3 h, 3–6 h, 6–24 h; single vs. serial) indicate that many omics signatures are anchored to early critical care sampling, typically at ICU admission or early in the ICU course, to support endotyping and diagnostic discrimination; some datasets also capture temporal progression. For example, genomic endotype studies commonly use admission blood gene expression profiles (Scicluna et al., 2017), whereas proteome-mapping studies can include repeated measures to describe proteomic changes over time (Mi et al., 2024). These designs allow omics signals to be interpreted as evolving trajectories rather than fixed snapshots (Davenport et al., 2016; Mi et al., 2024).

### 5.13 Comparator/reference standard (clinical criteria; Sepsis-3 shock definition; lactate; SOFA/qSOFA; other tools)

In diagnostic transcript panels, expert-judged clinical diagnosis (e.g., physician-panel consensus) and/or cohort-defined sepsis vs. non-infectious systemic inflammation are commonly used as reference standards (McHugh et al., 2015). For endotyping and prognostic omics, comparators are typically clinical phenotypes and severity measures (e.g., organ dysfunction, ICU characteristics) rather than replacement definitions of sepsis (Davenport et al., 2016; Scicluna et al., 2017). Septic shock is specified where Sepsis-3 operational criteria are used, including vasopressor requirement to maintain MAP ≥65 mmHg and lactate >2 mmol/L in the absence of hypovolemia (Singer et al., 2016).

### 5.14 Endpoints and prediction horizon (transition to vasopressor-dependent shock; time-to-shock; mortality as secondary)

In this category, mortality/outcome risk and biologically distinct response states (rather than time-to-shock progression) tend to be more often endpoint in this category. The 28-day mortality was related to the genomic endotypes (Mars1–4) (Scicluna et al., 2017), whereas the outcomes pertaining to prospective ICU cohorts were connected to the transcriptomic heterogeneity (Davenport et al., 2016). Proteomic mapping has been applied to discover etiologic, clinical phenotypic (organ failures), severity, and outcome-relevant subphenotypes (Mi et al., 2024). Transcript panels involved in diagnostics are focused on differentiating between sepsis and noninfectious systemic inflammation, and the performance can be measured using the ROC/AUC and incremental values in comparison to clinical variables (McHugh et al., 2015; Miller et al., 2018).

### 5.15 Validation approach (internal vs. external; calibration/transportability)

Compared with most single-protein biomarkers, the evidentiary strength for omics approaches is often higher because many studies use independent cohorts and/or multi-site designs. For example, SeptiCyte LAB includes both derivation and independent validation and has been tested across multiple sites and cohorts (McHugh et al., 2015; Miller et al., 2018). Genomic endotype categories have been confirmed in multi-ICU cohorts (Scicluna et al., 2017), and transcriptomic heterogeneity findings have been replicated in independent datasets (Davenport et al., 2016). Large integrative proteomic datasets enable broader phenotype discovery; however, bedside translation still requires platform standardization and prospective validation in the intended clinical workflow (Mi et al., 2024).

#### 5.15.1 Sepsis MetaScore (SMS)

Computed from vast datasets of transcriptomes, the Sepsis MetaScore (SMS) includes the expression of numerous immune and inflammatory genes. Such a composite score has been proven to discriminate sepsis from sterile inflammation as well as predict outcome. It has a great potential in early diagnosis of sepsis and in determination of mortality in critically painful patients (Farid and Abd El Hamed, 2021).

#### 5.15.2 FAIM3:PLAC8 Rrtio

The FAIM3:PLAC8 gene expression ratio has provided high diagnostic accuracy in the discrimination between bacterial and viral infections in febrile patients. Such a ratio might also allow the recognition of patients who are at a risk of developing a septic shock, and open the way to a new diagnosis of sepsis and a prediction stratification methodology.

#### 5.15.3 Proteomic markers

Sepsis proteomic studies have reported several proteins modified in patients with sepsis, disease severity, and organ dysfunction. Hemopexin, gelsolin and complement factors have been associated with immune activation, endothelial injury and metabolic dysfunction during sepsis. These proteins might be risk stratification markers and dictate personalized treatment strategies (Piccioni et al., 2021).

### 5.16 Metabolomic and lipidomic biomarkers

Sepsis stimulates a metabolic rewiring of host cells, which alters their energy metabolism, leads to oxidative stress and amino acid flux. Metabolomics, a tool that uses global profiling of small molecules, is generating new understanding of metabolic changes that occur in sepsis-induced shock.

### 5.17 Study design and setting (ED/ward/ICU; prospective vs. retrospective)

Legacy evidence arises from (i) prospective ED or hospital cohorts with early blood draws and outcome-stratified metabolomics, (ii) ICU septic shock cohorts, and (iii) randomized-trial biobanks that enable metabolomic sub-phenotyping with built-in external validation. For example, Seymour and colleagues analyzed the initial blood sample from an ED community-acquired pneumonia/sepsis cohort (GenIMS) using non-targeted mass spectrometry in an outcome-stratified case-control design (death within 90 days vs. survival) (Seymour et al., 2013). Metabolic sub-phenotypes in septic shock have also been derived and validated using blinded randomized controlled trial datasets (LeoPARDS derivation; VANISH validation) (Antcliffe et al., 2025). Severe septic shock-targeted metabolomics has additionally been explored in retrospective ICU biobanks (Ferrario et al., 2016).

### 5.18 Population characteristics (adult vs. pediatric; severity spectrum; inclusion criteria)

Most of the studies include cohorts of adult population fortified with severe sepsis and septic shock. Hospitalized adults who had 90-day survival was associated with metabolomics in GenIMS, which included CAP and sepsis (Seymour et al., 2013). On the one hand, severe shock ICU populations usually comprise those with high sequential organ failure assessment (SOFA) scores and/or hyperlactatemia; in a study by Ferrario et al., a subset of severe septic shock patients (SOFA >8; lactate >4 mmol/L) were considered (Ferrario et al., 2016). Randomized controlled trials are used to conduct sub-phenotyping in the patients recruited as soon as the septic shock occurs and/or those who need vasopressors (Antcliffe et al., 2025). Lipidomics has also been assessed in patients with sepsis caused by CAP in the ICU and has been compared to non-infected control groups in the ICU and outpatients (Chouchane et al., 2024).

### 5.19 Sampling window and kinetics-relevant timing (recognition; 0–3 h, 3–6 h, 6–24 h; single vs. serial)

The timing is also significantly different and can affect the interpretability. Other studies use an early sample (e.g., the first emergency-department draw) to measure physiology of presentation (Seymour et al., 2013). Others use serial sampling to outline trajectories: in LeoPARDS/VANISH, serum was sampled at enrollment and then at predetermined later timepoints (e.g., 24, 48–72, and approximately 96–120 h, dependent upon the trial) allowing analysis of stability and transition of clusters on metabolites between time. Paired early/late, ICU timepoints (e.g., day 1 and day 7 after shock diagnosis) have been used in targeted metabolomics to scale evolving lipid species and metabolites with 28- and 90-day mortality in severe septic shock (Ferrario et al., 2016).

### 5.20 Comparator/reference standard (clinical criteria; Sepsis-3 shock definition; lactate; SOFA/qSOFA; other tools)

Most metabolomic and lipidomic studies use established clinical definitions and severity scales as comparators rather than replacing them. Sepsis-3 definitions (vasopressor requirement to maintain MAP ≥65 mmHg with lactate >2 mmol/L despite adequate fluid resuscitation) remain the most common reference framework for septic shock (Singer et al., 2016). In practice, cohorts often combine SOFA/APACHE indices with lactate to contextualize metabolomic signatures; for example, severe shock metabolomics studies may add SOFA sub-scores and clinical variables to improve mortality models (Ferrario et al., 2016). Some lipidomics studies enroll Sepsis-3 patients within 24 h of ICU admission and use adjudicated clinical trajectories (rapid recovery vs. chronic critical illness/early death) as comparator phenotypes rather than a single physiologic cutoff (Sulaiman et al., 2024).

### 5.21 Endpoints and prediction horizon (transition to vasopressor-dependent shock; time-to-shock; mortality as a secondary outcome)

Numerous works are centered on mortality or poor-outcome phenotypes that are used in a clinical setting, and fewer explicitly estimate time-to-vasopressor shock. The model derivatives of GenIMS were meant to predict 90-day mortality off early emergency emergency-department sample (Seymour et al., 2013). The 28- and 90-day mortality were modeled using targeted features of ICU metabolomics, associating low lysophospholipid/phosphatidylcholine species and kynurenine-pathway to a difference in survival (Ferrario et al., 2016). Randomized controlled trial biobank work focuses on the persistence or trajectory of sub-phenotypes that predict mortality - in particular low-lysophospholipid phenotypes that are associated with increased mortality in both trial datasets (Antcliffe et al., 2025). Clinical endpoints (rapid recovery versus chronic critical illness/early death) have been linked to lipid mediator profiling, and certain ω −6 and 8 pathway mediators have been increased in poor -outcome groups (Sulaiman et al., 2024).

### 5.22 Validation approach (internal vs. external; calibration/transportability)

Validation quality across metabolomics studies is improving but remains heterogeneous. Stronger examples include cross-cohort validation of metabolomic sub-phenotypes, where clusters derived in one septic shock trial (LeoPARDS) were predicted and tested in an independent trial cohort (VANISH), providing a direct assessment of transportability (Antcliffe et al., 2025). Lipidomics studies are beginning to incorporate external validation cohorts to improve generalizability (Chouchane et al., 2024). Methodological work on meta-analytic cohort validation also addresses heterogeneity and replication challenges in metabolomics-based mortality prediction (Wang et al., 2020). Smaller pilot ICU studies should be interpreted as hypothesis-generating when they rely on internal modeling with limited sample sizes (Ferrario et al., 2016).

#### 5.22.1 Lactate/pyruvate ratios

The ratio of lactate to pyruvate forms a significant marker of tissue hypoxia and mitochondrial dysfunction during sepsis. High lactates are a classic marker of shock development, and the lactate/pyruvate index adds information on the severity of metabolic derangement in septic patients. Such a ratio can complement conventional biomarkers such as lactate and offer an improved measure of cellular energetics in the onset of the condition (Nesaragi and Patidar, 2020).

#### 5.22.2 Ketone bodies

Various elevations of ketone bodies, acetoacetate and beta-hydroxybutyrate, have been linked to metabolic stress and mitochondrial dysfunction in sepsis. Such molecules may act as the first indicators of tissue hypoxia and the failure of mitochondria, and both contribute to the development of sepsis-induced shock.

#### 5.22.3 Amino acid profiles

Amino acid profiling in sepsis reveals disturbed patterns of amino acid turnover; oxidative stress, cell damage and immune reprogramming. Such changes are capable of singling out patients with high risk for organ failure and the development of shock. Amino acid levels could also act as a guide to metabolic therapy and for nutritional support to the sepsis patients (Sutherland et al., 2011).

Advanced biomarkers such as microRNAs, circular RNAs, endothelial markers and proteomic profiles are revolutionizing our perspective regarding sepsis-instigated shock. These new biomarkers overcome the complexity of host response and incorporate inflammatory, immune, and metabolic changes. In addition to improved diagnostic achievement, they have a promising capacity for the early detection of sepsis, prognostic prognosis and personalized treatment approaches. With the advancement of the omics technologies, these novel biomarkers will be highly desirable in multimarker and clinical decision-support systems to enhance outcomes in sepsis-induced shock. Going forward, validation and subsequent clinical use of these biomarkers will be necessary to implement their use in a critical care environment (Agnello and Ciaccio, 2023).

## 6 Implementation and translation to clinical workflow

To be considered clinically actionable in the assessment of the risk of sepsis and septic shock, biomarkers should meet three useful criteria, including (i) timing (the results obtained should be available early enough to affect triage or risk increase); (ii) interpretability (results should be based on the validated cut-offs or risk categories of relevant populations); and (iii) value added (the results should be compared with those of currently applied clinical measurements and lactate values). Most single biomarkers seldom fulfill all three conditions; thus, in the near term, the most justifiable approach is the use of multimarkers panels in combination with clinical variables, to aid time-dependent decision-making, such as ICU referral, intensity of hemodynamic monitoring, and antimicrobial stewardship.

Biomarkers can only improve outcomes if they are translated into time-bounded clinical decisions that match sepsis physiology and ED/ICU workflows. In this review, “actionable” implementation means a biomarker result that (i) returns fast enough to affect a decision node, (ii) is interpretable for a defined endpoint and time window, and (iii) adds value beyond clinical assessment and routine testing. Using Sepsis-3 terminology, septic shock is defined by vasopressor requirement to maintain MAP ≥65 mmHg plus lactate >2 mmol/L despite adequate fluid resuscitation (Singer et al., 2016), and workflow recommendations should align with Surviving Sepsis Campaign guidance for early resuscitation and monitoring (Evans et al., 2021).

### 6.1 Clinical utility by decision node (0–3 h/3–6 h/6–24 h)

**FIGURE 4:**
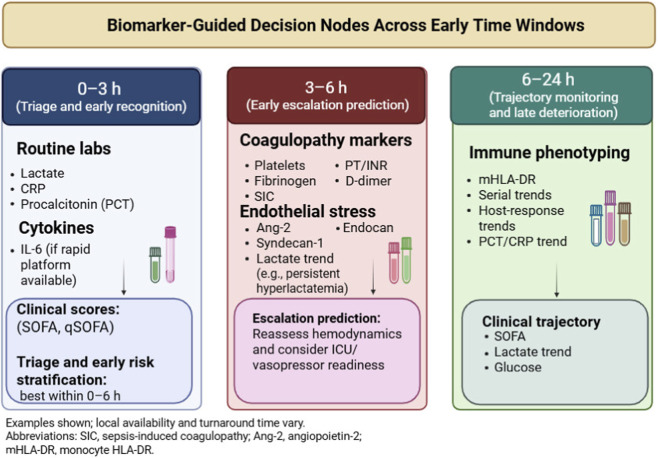
Biomarker-guided decision nodes across early time windows in suspected sepsis. The framework maps commonly available tests (0–3 h), escalation-oriented markers (3–6 h), and trajectory/immune phenotyping (6–24 h) to clinically actionable decisions (hemodynamic reassessment, ICU/vasopressor readiness, and detection of late deterioration). Serial trends (e.g., lactate, PCT/CRP) complement single time-point values. PCT, procalcitonin; SIC, sepsis-induced coagulopathy; Ang-2, angiopoietin-2; mHLA-DR, monocyte HLA-DR.

An overview of biomarker-guided decision nodes across early time windows in suspected sepsis is provided in Figure 4.

#### 6.1.1 Decision node 0–3 h: ED triage and early recognition

In the first hours, the main implementation goal is rapid recognition and risk tagging: identifying patients who appear “stable” but are likely to deteriorate. Biomarkers with the highest near-term feasibility are those already embedded in routine care (e.g., lactate, CRP, procalcitonin [PCT]) and those that can be measured rapidly on available platforms. Although inflammatory mediators (e.g., IL-6) are biologically attractive early markers, deployment depends on turnaround time and assay availability. In Sepsis-3 cohorts, IL-6, PCT, and lactate measured in the ED have shown discriminatory and prognostic performance for septic shock and short-term outcomes, supporting their role as early severity/escalation markers when aligned to clear endpoints and timing (Song et al., 2019). Because single biomarkers are rarely definitive across etiologies, their most defensible early use is as components of structured risk pathways (e.g., trigger earlier reassessment, senior review, and lower threshold for ICU evaluation), rather than as stand-alone “rules.”

#### 6.1.2 Decision node 3–6 h: Early escalation and shock transition risk

In the 3–6 h window, clinicians assess whether a patient is stabilizing with early resuscitation or progressing toward shock (e.g., rising vasopressor needs, persistent hyperlactatemia). Biomarkers used in this window should be evaluated primarily for short-horizon escalation prediction rather than for long-horizon mortality. Surveillance of thromboinflammation and endothelial injury can provide supportive risk information: coagulopathy phenotypes are common in sepsis and may coexist with vasoplegia and organ dysfunction (Iba and Levy, 2020). Endothelial markers (e.g., angiopoietin-2) reflect vascular leak/vasoplegia biology and may justify intensified monitoring and earlier escalation readiness, but they should not replace bedside hemodynamic evaluation (Zhuo et al., 2024).

#### 6.1.3 Decision node 6–24 h: Trajectory monitoring and late deterioration

Sepsis is dynamic; thus, most of the biomarkers prove to be most informative when evaluated as trajectories, instead of single snapshot. Monitoring of serial changes in host-response markers (ex various PCT/CRP dynamics) can be used to justify re-evaluation of unsatisfactory source management, resistant pathogen, or complication (Meisner, 2002). Immune suppression phenotyping can also imply clinically in some specific environments at this stage. Immunosuppression caused by sepsis is a known biological stage that can lead to secondary infections and delayed worsening (Hotchkiss et al., 2013). Monocytes HLA-DR (mHLA-DR) is categorized as a biomarker which can detect immunosuppressed phenotype but the methods should be standardized by flow cytometry and the thresholds should be clearly defined (Quadrini et al., 2021). In case of limited access to sampling or labs, tube stabilization may increase feasibility, but it also could lead to systematic changes in measurements that need to be considered in clinical thresholds (Hamada et al., 2022).

### 6.2 Analytical validity and standardization

One of the main obstacles to the widespread use of biomarker-based methods is the resulting variability in threshold definitions and performance methods due to assay system differences, specimen handling procedures, and clinical situations. This is particularly relevant with immune phenotyping and cytokine assays, in which pre-analytical time, and platform heterogeneity can lead to a high degree of variance and with multiplex analyte panels, in which batch effects add to the interpretation problem. Standardized cytometric standards and consensus threshold settings are thus the requirements of transportability of immune suppression biomarkers (Quadrini et al., 2021). Mechanistic coherence is strong, in relation to the functional immune systems, i.e., endotoxin tolerance, but at the bedside, standardization of the stimulation procedure, standard response units, and high-quality control measures are required (Cavaillon and Adib-Conquy, 2006). It is impossible to achieve a reliable multi-center performance or implement a decision support system that can extrapolate between clinical institutions, without explicit analytical standardization.

### 6.3 Workflow, turnaround time, and point-of-care feasibility

For early shock prediction at presentation, clinical utility depends on whether results are available within the ED resuscitation window (typically 0–2 h). Routine laboratory markers and standard coagulation panels are already accessible in most hospitals. Endothelial markers, however, are more often measured using ELISA or specialized immunoassays and are not yet part of routine ED/ICU workflows (Zhuo et al., 2024). Immune phenotyping of mHLA-DR requires flow cytometry infrastructure and is highly sensitive to specimen handling constraints (Quadrini et al., 2021; Hamada et al., 2022). Newer host-response transcriptomic technologies can deliver results within ∼1 h on cartridge-based systems, but they still require high-quality external validation against short-horizon shock endpoints and careful integration into clinical processes (Balk et al., 2024). The table below summarizes the clinical readiness across biomarker categories (Table 5).

**TABLE 5:** Clinical readiness ranking of biomarker categories for early shock prediction at presentation.

Biomarker category	Primary intended use case	Optimal decision window	Typical platform/turnaround time	External validation strength^a^	Practical readiness (ED/early ward)
Routine laboratory and coagulation panels (e.g., lactate, CBC indices, PT/INR, platelets, D-dimer)	Immediate triage support; early escalation and monitoring intensity	0–3 h (serial as needed)	Routine hospital analyzers; ∼15–60 min	Strong	High
Acute-phase proteins (e.g., PCT, CRP)	Early risk stratification; adjunct to clinical scores	0–3 h to 3–6 h (repeat for trends)	Standard immunoassays; ∼30–120 min	Moderate	High–Medium
Inflammatory cytokines/mediators (e.g., IL-6, IL-8, TNF-α)	Physiologic severity profiling; adjunct prognostic enrichment	0–3 h to 6–24 h (kinetics-dependent)	ELISA/chemiluminescence platforms; often >2 h (variable)	Moderate	Medium
Endothelial/glycocalyx injury markers (e.g., Ang-2, sTM, syndecan-1)	Microvascular injury/endotheliopathy risk; escalation monitoring	3–6 h to 6–24 h (often serial)	Specialized ELISA; not routine; hours–days	Weak	Low
Immune suppression phenotyping (e.g., mHLA-DR trajectories; functional immune assays)	Trajectory monitoring; late deterioration/secondary infection risk	6–24 h and beyond	Flow cytometry/functional assays; infrastructure-dependent; hours	Weak (for presentation-time prediction)	Low
Host-response transcriptomics/multi-gene signatures (cartridge-based systems)	Rapid host-state classification; early escalation support (emerging)	0–3 h (if point-of-care)	Cartridge-based platforms; ∼45–90 min (site-dependent)	Moderate (emerging)	Medium

^a^ Strength definitions are provided immediately above (Strong/Moderate/Weak external validation for early shock prediction at presentation).

Definition used in Table 5 (External validation for early shock prediction at presentation): Strong = validated in an independent external cohort and/or multi-site prospective study using a short-horizon shock endpoint (e.g., Sepsis-3 septic shock or vasopressor initiation within a defined window) with transparent performance reporting; Moderate = evaluated in an external cohort or multi-site study but with limited endpoint/horizon matching and/or incomplete calibration reporting; Weak = internal validation only (single cohort/site) or no independent validation.

### 6.4 Clinical validity and external validation

To establish clinical validity for early shock prediction, studies should clearly define (i) sampling time, (ii) the endpoint (typically Sepsis-3 septic shock), and (iii) the comparator (e.g., clinical scores, lactate, hemodynamic variables). Many biomarkers are strongly associated with mortality or organ dysfunction, but far fewer have been validated for short-horizon shock escalation. Evidence for endothelial and immune biomarkers is often prognostic; therefore, predictor claims should be limited to studies in which endpoints and sampling timelines match the intended bedside use (Zhuo et al., 2024; Quadrini et al., 2021). For omics-based platforms, broad generalizability also depends on external validation across institutions and heterogeneous case mixes, ideally in prospective cohorts (Balk et al., 2024).

### 6.5 Clinical utility, interventional evidence, and cost-effectiveness

The move towards clinical implementation even with an established analytical and clinical validity needs to be informed by evidence that biomarker-based pathways can change therapeutic decisions and improve patient outcomes. An implementation framework with a strong emphasis on feasibility markers, such as routine laboratory tests and coagulation tests, with a standardized clinical process could be described pragmatically as follows: (i) the primary focus of influencing the implementation of high-feasibility markers, including inflammatory, endothelial, and coagulation markers as long as the assays are within the turnaround time that facilitates a timely decision-making process; (ii) the addition of biologically complementary category, including inflammatory, endothelial, and coagulation.

### 6.6 Regulatory adoption, ethics, and interpretive safeguards

Because regulatory approval, data privacy, and interpretive safeguards determine real-world feasibility, these issues are discussed in detail in Sections 6.5, 6.6. Workflow implementation, turnaround time, and validation needs are addressed in Sections 6.3, 6.4. Table 5 ranks biomarker categories by practical readiness for early prediction of Sepsis-3 septic shock at presentation (considering optimal time of use, serial testing, assay platform, and feasibility/turnaround), consistent with the need for rapid and scalable bedside decision support (Singer et al., 2016; Evans et al., 2021). The most deployable groups are routine laboratory and coagulation assays with short turnaround times, whereas endothelial markers and immune suppression phenotyping remain constrained by assay standardization and platform availability (Iba and Levy, 2020; Quadrini et al., 2021; Hamada et al., 2022; Zhuo et al., 2024). Emerging host-response transcriptomic technologies can approach point-of-care turnaround, but they still require high-quality external validation specifically against short-horizon shock endpoints at presentation (Balk et al., 2024).

## 7 Future directions

Sepsis-induced shock management is changing with the advent of biomarker discovery, omics technologies, and devices for clinical decision-making. Despite advancements, factors, including standardization, cost, and timing, limit the biomarker utility. Integration into clinical practice is the future and will depend on overcoming these barriers through multidisciplinary research, new technologies, and regimes (Venter et al., 2010).

### 7.1 Multimarker panels and integrated biomarker systems

Combining multimarker panels using biomarkers for various biological pathways offers a more comprehensive sepsis evaluation. Panels that combine IL-6, PCT, CRP and new markers such as microRNAs and circular RNAs will increase diagnostic accuracy and individual treatment approaches. Combined systems integrating biological data/clinical variables provide real-time patient condition observations (Reinhart et al., 2012).

### 7.2 Machine learning and artificial intelligence

With large datasets, AI and ML can predict the continuation of sepsis and shock probability, as well as responses to treatment. Such technologies counter clinical variability and can support automated biomarker analysis, providing on-time, point-of-care diagnostics in managing sepsis (Ke et al., 2023).

### 7.3 Personalized medicine and immunomodulatory therapies

So, tailored therapies based on the data from the biomarkers will intervene in immune dysregulation in sepsis. Immunomodulatory therapy involving immune checkpoint inhibitors and cytokine modification utilised with personalised antibiotic stewardship optimizes sepsis management and resistance and enhances outcomes (von Groote and Meersch-Dini, 2022).

### 7.4 Global accessibility and resource-limited settings

A critical intervention for global accessibility is using low-cost, rapid biomarker assays for point-of-care testing. Microfluidic devices, biosensors and lateral flow immunoassays can help home country patients and patients overseas in low-resource settings receive timely sepsis diagnosis and treatment with affordable, portable devices (Turgman et al., 2023).

### 7.5 Regulatory and clinical validation

Multicenter clinical validation is critical for biomarker adoption related to sepsis by the regulators and on a broader scale. Large-scale RCTs will help prove the efficacy and cost-efficiency of biomarker panels so that the novel biomarkers and diagnoses reach clinical practice promptly (Al Jalbout et al., 2019).

### 7.6 Ethical considerations and data privacy

Given the influence of biomarkers and AI on clinical practice, informed consent and patient autonomy are the most important aspects. Data privacy issues, such as those associated with sensitive health information, must be resolved for the sake of following policies, such as HIPAA and GDPR, to maintain the confidentiality of patients (Shachar et al., 2023).

## 8 Conclusion

Introducing the biomarkers into the clinical examination of sepsis-induced shock can completely shift the paradigm for early diagnosis and risk stratification, as well as the development of targeted therapies. Biomarkers represent a strong diagnostic and prognostic foundation in describing the reasons behind sepsis pathophysiology and reveal possibilities for targeted intervention, including immunomodulating therapies and precision medicine strategies. The clinical utility of biomarkers in sepsis is still hampered by tremendous challenges such as biological variability, cost, lack of standardization, and analytical complexity. Future studies are necessitated by the need to create multimarker panels, machine learning algorithms and low-cost, transportable assays that are used in point of care testing, making them available in areas of limited resources. Furthermore, multicenter clinical trials and regulatory architectures must validate these biomarkers and define their place in routine clinical work. The further development of the sepsis markers research, cooperative actions to overcome these problems will be the key to success in the improvement of diagnosis of sepsis, early intervention, and the outcomes of the patients, saving the lives and health of the patients with sepsis-induced shock.
